# Supplementary material for: Tuberculosis notifications in regional Victoria, Australia: Implications for public health care in a low incidence setting
Source: PLoS One. 2023 Mar 21;18(3):e0282884. doi: 10.1371/journal.pone.0282884 (PMC10030020; doi:10.1371/journal.pone.0282884)
Supplement: Attachment — Submitted filename: PONE-D-22-00893_R2_comments.pdf [file pone.0282884.s004.pdf]

PLOS ONE

Tuberculosis notifications in regional Victoria, Australia: implications for public health care in a low incidence setting

--Manuscript Draft--

**Manuscript Number:**	PONE-D-22-00893R2
**Article Type:**	Research Article
**Full Title:**	Tuberculosis notifications in regional Victoria, Australia: implications for public health care in a low incidence setting
**Short Title:**	Tuberculosis in regional Victoria
**Corresponding Author:**	Nompilo Moyo, PhD Candidate<br>La Trobe University - Bundoora Campus: La Trobe University<br>Bundoora, Victoria AUSTRALIA
**Keywords:**	Keywords: regional, metropolitan, tuberculosis, treatment completion, delayed diagnosis
**Abstract:**	Background: Regionality is often a significant factor in tuberculosis (TB) management and outcomes worldwide. A wide range of context-specific factors may influence these differences and change over time. We compared TB treatment in regional and metropolitan areas, considering demographic and temporal trends affecting TB diagnosis and outcomes. Methods: Retrospective analyses of data for patients notified with TB in Victoria, Australia, were conducted. The study outcomes were treatment delays and treatment outcomes. Multivariable Cox proportional hazard model analyses were performed to investigate the effect of regionality in the management of TB. Six hundred and eleven (7%) TB patients were notified in regional and 8,163 (93%) in metropolitan areas between 1995 and 2019. Of the 611 cases in the regional cohort, 401 (66%) were overseas-born. Fifty-one percent of the overseas-born patients in regional Victoria developed TB disease within five years of arrival in Australia. Four cases of multidrug-resistant tuberculosis were reported in regional areas, compared to 97 cases in metropolitan areas. A total of 3,238 patients notified from 2012 to 2019 were included in the survival analysis. Patient follow-up was censored at the first visit to the health care facility (Patient treatment delay) and at the initiation of TB treatment (Health system delay). Patient, health system, and treatment delays were similar in regional and metropolitan areas for cases with pulmonary involvement. Cases with extrapulmonary TB in regional areas have a non-significantly longer healthcare system delay than patients in metropolitan (median 64 days versus 54 days, AHR = 0.8, 95% CI 0.6-1.0, P = 0.094).<br>Conclusion: Tuberculosis in regional Victoria is common among the overseas-born population, and patients with extrapulmonary TB in regional areas experienced a non-significant minor delay in treatment commencement with no apparent detriment to treatment outcomes. Improving access to LTBI management in regional areas may reduce the burden of TB.
**Order of Authors:**	Nompilo Moyo, PhD Candidate
	Ee Laine T Tay
	James Trauer
	Leona Burke
	Justin Jackson
	Robert Commons
	Sarah Boyd
	Kasha Singh
	Justin Denholm
**Response to Reviewers:**	Tuberculosis notifications in regional Victoria, Australia: implications for public health care in a low incidence setting

Powered by Editorial Manager® and ProduXion Manager® from Aries Systems Corporation

Response to reviewer 2
Your feedback 1. The Methods section still requires revisions. Currently there are sub-sections which are not properly described. For example, the study design sub-section is just one short sentence. I suggest revising this section to include the following sub-sections in this particular order: Data Source, Study Population, Variables, and Statistical Analysis. Under the Data Source sub-section - describe the data source, study design and study setting. Under Study Population sub-section - describe the target population by age, inclusion and exclusion criteria and missing data. Under the Variables sub-section - describe the variables, dependent and independent variables. Under Statistical Analysis sub-section - describe the analyses conducted in the study.

Our response. We have organised the methods section under the suggested sub-sections. See revised lines 99 to 167.

Response to reviewer 3
Your feedback 1: Lines 30-32: the methods section of the abstract is quite brief missing important details that will help readers to understand the results presented from a survival analysis. Sample size, follow-up, date variables, outcomes, censoring, and statistical analysis issues are missing.

Our response. We have amended the abstract, and it now reads, "Background: Regionality is often a significant factor in tuberculosis (TB) management and outcomes worldwide. A wide range of context-specific factors may influence these differences and change over time. We compared TB treatment in regional and metropolitan areas, considering demographic and temporal trends affecting TB diagnosis and outcomes.
Methods: Retrospective analyses of data for patients notified with TB in Victoria, Australia, were conducted. The outcomes were treatment delays and treatment outcomes. Multivariable Cox proportional hazard model analyses were performed to investigate the effect of regionality in the management of TB. Six hundred and eleven (7%) TB patients were notified in regional and 8,163 (93%) in metropolitan areas between 1995 and 2019. Of the 611 cases in the regional cohort, 401 (66%) were overseas-born. Fifty-one percent of the overseas-born patients in regional Victoria developed TB disease within five years of arrival in Australia. Four cases of multidrug-resistant tuberculosis were reported in regional areas, compared to 97 cases in metropolitan areas. A total of 3,238 patients notified from 2012 to 2019 were included in the survival analysis. Patient follow-up was censored at the first visit to the health care facility (Patient treatment delay) and at the initiation of TB treatment (Health system delay). Patient, health system, and treatment delays were similar in regional and metropolitan areas for cases with pulmonary involvement. Cases with extrapulmonary TB in regional areas have a non-significantly longer healthcare system delay than patients in metropolitan (median 64 days versus 54 days, AHR = 0.8, 95% CI 0.6-1.0, P = 0.094).
Conclusion: Tuberculosis in regional Victoria is common among the overseas-born population, and patients with extrapulmonary TB in regional areas experienced a non-significant minor delay in treatment commencement with no apparent detriment to treatment outcomes. Improving access to LTBI management in regional areas may reduce the burden of TB." Lines 26-49.

Your feedback 2. Line 36-39-->the statement in these lines feels like labeling. In the absence of adequate number of cases, it is difficult to associate multidrug resistant TB and being overseas born. Furthermore, a statement in line 36 reads, 'the proportion of MDR-TB cases in regional vs metropolitan areas is similar'. In the next line, however, it presents only four cases of MDR-TB in regional vs 97 in metropolitan. The statements in the lines indicated above are difficult to followpresent them consistently in terms of proportion or in absolute numbers. The data presented in Table 1 of the body of the document, do not support this statement. 4 MDR-TB vs 0 in regional and metropolitan--> this data is not adequately powered to support the statement provided in these lines.

Our response. We have amended the statement and it now reads "Four cases of multidrug-resistant tuberculosis were reported in regional areas, compared to 97 cases in metropolitan areas." Lines 37-38.

Your feedback 3. Lines 40-44: "Cases with extra pulmonary TB in regional areas have a non-significantly longer healthcare system delay than patients in metropolitan

Powered by Editorial Manager® and ProduXion Manager® from Aries Systems Corporation

(median 64 days versus 54 days, AHR = 0.8, 95% CI 0.6-1.0, P = 0.094). People living in regional areas have a non-significantly higher odds of dying of TB (AOR = 1.8, 95% CI 0.7-4.2, P = 0.198)."
In the above text, the authors presented mixed effect sizes, AHR vs AOR. However, the effect estimate from the Cox proportional hazards region is expressed in HRs than ORs. The other thing is that the authors should clearly specify their outcome of interest than generally providing 'TB mgt. outcome'.

Our response. We have amended the abstract, please refer to our response 1. Lines 26-49.

Your feedback 4. The other thing is that the authors should clearly specify their outcome of interest than generally providing 'TB mgt. outcome'.

Our response. We have added the following statement to the abstract and methods sections: “The study outcomes were treatment delays and treatment outcomes”. Lines 31, 144-145.

Your feedback 5. While information presented in the background is critical to understand context of the problem, its nature, efforts to reduce the extent of the problem, challenges, gaps, and the need to conduct the current study, it is only presented in 18 lines missing important details. Therefore, i suggest the authors to consider adding a few details to give insight to the problem studied.

Our response. Thank you for this suggestion. We have added the following statement: “Understanding TB treatment delays among regional patients provides important insights into Victorian TB programme performance and is a critical step towards tuberculosis elimination. Globally, TB surveillance data have been recognised as an important data source for assessing the disease burden and epidemiological trends in TB (World Health Organization, 2022). Evaluating treatment outcomes and delays in regional areas will inform practice and policy.” Lines 90-95.

Your feedback 6. Data analysis- Line 139: consider here too the comments provided in the abstract regarding data analysis. Lines 150-153: present the global test results and also for the independent variables to attest that the proportional hazard regression was met.

Our response. We have amended the data analysis, and it now reads: “Descriptive and multivariable analyses were performed. Incidence rates were calculated using the mid-year estimated resident population. Pearson’s x2 test was used to test the association between categorical variables. A two-tailed p-value of <0.05 was considered statistically significant. In logistic regression, we compared complete treatment with death, irrespective of the cause, lost to follow-up and transferred interstate or overseas. Died of TB was compared with completed treatment, lost to follow-up, died of other causes during treatment for TB, and transferred interstate or overseas. We included all independent variables in all multivariable analyses because we believed they could all affect the outcomes. However, our variable of interest was regionality. The proportional-hazards assumption was assessed using Kaplan-Meier survival curves by including time-dependent covariates in the model and with Schoenfeld residuals. In cases where proportionality assumptions were not met, analyses were stratified. Kaplan-Meier survival curves were used to show various delays in presentation, diagnosis, and treatment between regional and metropolitan cohorts, and Cox proportional hazard analyses were performed to assess these delays. Patient follow-up was censored, 1. at the first visit to a health care facility (Patient treatment delay), 2. at the initiation of TB treatment (Health system delay), 3. at the time a chest x-ray was performed (Diagnostic delay), 4. at the treatment initiation (Treatment initiation delay). Because of limited previous data, analyses of treatment outcomes were conducted using data from 2005 to 2019, while analyses of treatment delays used data from 2012 to 2019.” Lines 190-210.

Your feedback 7. Results- Line 158: the 7% and 93% reported cases of TB do not reflect that 45 cases did not have residential information regarding their affiliation to regional of metropolitan.

Powered by Editorial Manager® and ProduXion Manager® from Aries Systems Corporation

	Our response: We have amended this statement, and now it reads; "A total of 8,819 TB cases were notified to the Victorian Government Department of Health between 1995 and 2019. Among the 8,819 cases, 611 (7%) were recorded in regional areas, 8,163 (93%) in metropolitan areas of Victoria and 45 (1%) had neither regional nor metropolitan residential addresses (see Fig 1). Forty-five cases with no residential addresses were excluded from the study as they were classified as neither regional nor metropolitan." Lines 215-219.<br><br>Your feedback 8. Line 164-65: it is good to present the number of cases excluded. Or preferably provide the progress of pts. in a flow diagram.<br><br>Our response. Thank you for this suggestion. We have now provided the flow of patients through the study, see Fig 1. Line 224.<br><br>Your feedback 9. Line 175-177: this result has not been well reflected in the abstract.<br><br>Our response. Lines 175-177 were referring specifically to issues related to missing data. However, the abstract has been amended to more clearly reflect the overall findings as outlined above. Lines 26-49.<br><br>Your feedback 10. Table 1: the font size of contents of the table is significantly different from the text in the body.<br><br>Our response. We have increased the font size on all tables to 11 points.<br><br>Your feedback 11. The authors also consider avoiding in places where the total add to hundred or cell values added to the sample in respective subgroup. Or consider using '0' or NA to represent 'not available'<br><br>Our response. The tables have been amended accordingly.<br><br>Your feedback 12. Discussion- Owning to the arrival of overseas born individuals from high TB burden countries to Australia, there could be an active search for TB among this particular group which may introduce a diagnostic suspicion bias. Was there an effort in this study to exclude that diagnostic suspicion bias was not an issue or was there an effort to reduce it if there was any?<br><br>Our response. Thanks for raising this point, which we agree may be important in many contexts. We do not believe that this is a significant issue in our study, as the considerable majority of migration-associated testing for TB an active case finding occurs prior to visa issuing in countries of origin and are thus not reflected in these TB cases presented here. Overall, then, we do not account further for diagnostic suspicion bias during the study period.<br><br>Reference<br>World Health Organisation. (2022). Strengthening TB surveillance. World Health Organisation. https://www.who.int/westernpacific/activities/strengthening-tb-surveillance
**Additional Information:**	
**Question**	**Response**
**Financial Disclosure**<br><br>Enter a financial disclosure statement that describes the sources of funding for the work included in this submission. Review the submission guidelines for detailed requirements. View published research articles from *PLOS ONE* for specific examples.	The authors received no specific funding for this work.

This statement is required for submission and **will appear in the published article** if the submission is accepted. Please make sure it is accurate.<br><br>**Unfunded studies**<br>Enter: *The author(s) received no specific funding for this work.*<br><br>**Funded studies**<br>Enter a statement with the following details:<br>• Initials of the authors who received each award<br>• Grant numbers awarded to each author<br>• The full name of each funder<br>• URL of each funder website<br>• Did the sponsors or funders play any role in the study design, data collection and analysis, decision to publish, or preparation of the manuscript?<br>• **NO** - Include this sentence at the end of your statement: *The funders had no role in study design, data collection and analysis, decision to publish, or preparation of the manuscript.*<br>• **YES** - Specify the role(s) played.<br><br>* typeset	
**Competing Interests**<br><br>Use the instructions below to enter a competing interest statement for this submission. On behalf of all authors, disclose any competing interests that could be perceived to bias this work—acknowledging all financial support and any other relevant financial or non-financial competing interests.<br><br>This statement is required for submission and **will appear in the published article** if the submission is accepted. Please make sure it is accurate and that any funding sources listed in your Funding Information later in the submission form are also declared in your Financial Disclosure statement.<br><br>View published research articles from *PLOS ONE* for specific examples.	The authors have declared that no competing interests exist.

**NO authors have competing interests**<br><br>Enter: *The authors have declared that no competing interests exist.*<br><br>**Authors with competing interests**<br><br>Enter competing interest details beginning with this statement:<br><br>*I have read the journal's policy and the authors of this manuscript have the following competing interests: [insert competing interests here]*<br><br>* typeset	
**Ethics Statement**<br><br>Enter an ethics statement for this submission. This statement is required if the study involved:<br><br>• Human participants<br>• Human specimens or tissue<br>• Vertebrate animals or cephalopods<br>• Vertebrate embryos or tissues<br>• Field research<br><br>Write "N/A" if the submission does not require an ethics statement.<br><br>General guidance is provided below. Consult the submission guidelines for detailed instructions. **Make sure that all information entered here is included in the Methods section of the manuscript.**	Approval from a Human Research Ethics Committee for this study was not required as the data were collected for the purposes of public health action, as defined in the Public Health and Wellbeing Act 2008 and was considered as being for quality assurance and auditing purposes. Patients were informed of the purpose of data collection and consented to their data being used for tuberculosis surveillance and medical research at the time of collection. All data were fully anonymized during the data extraction process. For example, names, phone numbers, addresses, dates of birth of participants were removed. Patient identification numbers, postcodes, gender were coded, and age was changed to age group. In the publications that come from this study, patients will remain anonymous.

Powered by Editorial Manager® and ProduXion Manager® from Aries Systems Corporation

**Format for specific study types**<br><br>**Human Subject Research (involving human participants and/or tissue)**<br>• Give the name of the institutional review board or ethics committee that approved the study<br>• Include the approval number and/or a statement indicating approval of this research<br>• Indicate the form of consent obtained (written/oral) or the reason that consent was not obtained (e.g. the data were analyzed anonymously)<br><br>**Animal Research (involving vertebrate animals, embryos or tissues)**<br>• Provide the name of the Institutional Animal Care and Use Committee (IACUC) or other relevant ethics board that reviewed the study protocol, and indicate whether they approved this research or granted a formal waiver of ethical approval<br>• Include an approval number if one was obtained<br>• If the study involved *non-human primates*, add *additional details* about animal welfare and steps taken to ameliorate suffering<br>• If anesthesia, euthanasia, or any kind of animal sacrifice is part of the study, include briefly which substances and/or methods were applied<br><br>**Field Research**<br><br>Include the following details if this study involves the collection of plant, animal, or other materials from a natural setting:<br>• Field permit number<br>• Name of the institution or relevant body that granted permission	
**Data Availability**<br><br>Authors are required to make all data underlying the findings described fully available, without restriction, and from the time of publication. PLOS allows rare exceptions to address legal and ethical concerns. See the PLOS Data Policy and FAQ for detailed information.	Yes - all data are fully available without restriction

Powered by Editorial Manager® and ProduXion Manager® from Aries Systems Corporation

A Data Availability Statement describing where the data can be found is required at submission. Your answers to this question constitute the Data Availability Statement and **will be published in the article**, if accepted.<br><br>**Important:** Stating 'data available on request from the author' is not sufficient. If your data are only available upon request, select 'No' for the first question and explain your exceptional situation in the text box.<br><br>Do the authors confirm that all data underlying the findings described in their manuscript are fully available without restriction?	
**Describe where the data may be found in full sentences. If you are copying our sample text, replace any instances of XXX with the appropriate details.**<br><br>• If the data are **held or will be held in a public repository**, include URLs, accession numbers or DOIs. If this information will only be available after acceptance, indicate this by ticking the box below. For example: *All XXX files are available from the XXX database (accession number(s) XXX, XXX.*).<br>• If the data are all contained **within the manuscript and/or Supporting Information files**, enter the following: *All relevant data are within the manuscript and its Supporting Information files.*<br>• If neither of these applies but you are able to provide **details of access elsewhere**, with or without limitations, please do so. For example:<br><br>*Data cannot be shared publicly because of [XXX]. Data are available from the XXX Institutional Data Access / Ethics Committee (contact via XXX) for researchers who meet the criteria for access to confidential data.*<br><br>*The data underlying the results presented in the study are available from (include the name of the third party*	***PA @ ACCEPT: ask for Dryad DOI***<br>Data will be available on this website: https://datadryad.org/stash

Powered by Editorial Manager® and ProduXion Manager® from Aries Systems Corporation

and contact information or URL).<br>• This text is appropriate if the data are owned by a third party and authors do not have permission to share the data.<br><br>* typeset	
Additional data availability information:	

Powered by Editorial Manager® and ProduXion Manager® from Aries Systems Corporation

Manuscript

Click here to access/download;Manuscript;Tuberculosis_R2_Clean copy.docx

Tuberculosis notifications in regional Victoria, Australia: implications for public health care in a low incidence setting

Moyo N[1, 2*], Tay EL[3], Trauer JM[1, 4] Burke L[1], Jackson J[5, 6], Commons RJ[7, 8], Boyd SC[9], Singh KP[10], Denholm JT[1, 10]

[1] Victorian Tuberculosis Program, Melbourne Health, Melbourne, Victoria, Australia

[2] La Trobe University, School of Nursing and Midwifery, Melbourne, Victoria, Australia

[3] Communicable Diseases Epidemiology and Surveillance Unit, Health Protection Branch, Public Health Division, Department of Health, Victoria, Australia

[4] Monash University, School of Public Health and Preventive Medicine, Melbourne, Victoria, Australia

[5] Department of Medicine, Albury-Wodonga Health, Wodonga, Victoria, Australia

[6] Faculty of Medicine, University of New South Wales Rural Clinical School, Albury Campus, New South Wales, Australia

[7] Internal Medicine Services, Ballarat Health Services, Ballarat, Victoria, Australia

[8] Global and Tropical Health Division, Menzies School of Health Research and Charles Darwin University, Darwin, Northern Territory, Australia

[9] Royal Brisbane and Women's Hospital, Brisbane, South Australia, Australia

[10] Department of Infectious Diseases, Doherty Institute for Infection and Immunity, University of Melbourne, Parkville, Victoria, Australia

* nompiloo@yahoo.com

Abstract

Background: Regionality is often a significant factor in tuberculosis (TB) management and outcomes worldwide. A wide range of context-specific factors may influence these differences and change over time. We compared TB treatment in regional and metropolitan areas, considering demographic and temporal trends affecting TB diagnosis and outcomes. Methods: Retrospective analyses of data for patients notified with TB in Victoria, Australia, were conducted. The study outcomes were treatment delays and treatment outcomes. Multivariable Cox proportional hazard model analyses were performed to investigate the effect of regionality in the management of TB. Six hundred and eleven (7%) TB patients were notified in regional and 8,163 (93%) in metropolitan areas between 1995 and 2019. Of the 611 cases in the regional cohort, 401 (66%) were overseas-born. Fifty-one percent of the overseas-born patients in regional Victoria developed TB disease within five years of arrival in Australia. Four cases of multidrug-resistant tuberculosis were reported in regional areas, compared to 97 cases in metropolitan areas. A total of 3,238 patients notified from 2012 to 2019 were included in the survival analysis. Patient follow-up was censored at the first visit to the health care facility (Patient treatment delay) and at the initiation of TB treatment (Health system delay). Patient, health system, and treatment delays were similar in regional and metropolitan areas for cases with pulmonary involvement. Cases with extrapulmonary TB in regional areas have a non-significantly longer healthcare system delay than patients in metropolitan (median 64 days versus 54 days, AHR = 0.8, 95% CI 0.6-1.0, P = 0.094).

Conclusion: Tuberculosis in regional Victoria is common among the overseas-born population, and patients with extrapulmonary TB in regional areas experienced a non-significant minor delay in treatment commencement with no apparent detriment to

treatment outcomes. Improving access to LTBI management in regional areas may reduce the burden of TB.

Keywords: regional, metropolitan, tuberculosis, treatment completion, delayed diagnosis

Word count: 3,715 words, excluding tables and references.

Background

Victoria is the second most highly populated state in Australia, with 6.69 million residents as of March 2020 [1]. It has the highest population growth rate (1.8%), with net overseas migration functioning as the primary contributor to population growth in Victoria [1].

Victoria is divided into two distinct socio-geographic areas, metropolitan and regional. Metropolitan is defined as the 31 local government areas of the city of Melbourne, while the 48 local government areas outside of the city are defined as regional. Based on 2016 census data, 4,485,211 of Victoria's total population of 5,926,624 lived within Melbourne, with less than 25% of the population living regionally [2]. This equates to an average population density of 500 people per square kilometre in metropolitan Victoria compared to an average of 6 people per square kilometre in regional Victoria.

There are some differences in the provision of health care services between Victoria's metropolitan and regional areas. Unlike patients in most metropolitan areas, patients in regional Victoria may reside a considerable distance from hospitals. Most regional hospitals do not have negative pressure rooms for isolating TB patients during their infectious stage; therefore, infectious patients need to be transferred to metropolitan hospitals. Some regional hospitals have no on-site TB specialist medical practitioners, and

because of the distance from the hospital, there are very few opportunities for home visits from Victorian Tuberculosis Program specialist nursing staff, so regional patients must rely on telephone or video consultations for their diagnostic and follow-up consultations as well as their treatment supervision visits. The Victorian Tuberculosis Program (VTP) is Victoria's state-wide provider and coordinator of tuberculosis control.

In regional Victoria, the number of people born overseas is increasing. For example, in the 2006 census, there were 1,964 Indian-born people recorded as living in regional Victoria, which grew to 8,592 persons in 2016; similarly, the Philippines-born population was 2,700 in 2006 and 6,085 in 2016 [2]. The Australian Government has made changes to Commonwealth immigration policy intended to stimulate economic growth outside metropolitan areas in recent years. These various changes are focused on attracting migrants and international students to regional areas [3]. Historically, most migrants to Victoria have settled in the capital city of Melbourne, with many coming from countries with high TB incidence, such as India, the Philippines, and Sudan [4,5]. Such changes to policy influence migration patterns and may impact the distribution of TB cases within Victoria, which may also have implications for optimising health service delivery models [5].

TB incidence in Victoria remains low, with 436 TB cases notified in 2018, representing 6.9 cases per 100,000 population [6]. In Australia, research to date has tended to focus on metropolitan areas, where case numbers typically predominate. Understanding TB treatment delays among regional patients provides important insights into VTP performance and is a critical step towards tuberculosis elimination. Globally, TB surveillance data have been recognised as an important data source for assessing the disease burden and epidemiological trends in TB [7]. Evaluating treatment outcomes and delays in regional

areas will inform practice and policy. We aimed to describe notified TB cases in regional Victoria from 1995 to 2019, including trends and outcomes over these 24 years.

Methods

Data Source

We used routinely collected TB surveillance data. Data for all notified active tuberculosis cases in Victoria are collected by the VTP nurse consultants. Data are stored electronically in the Public Health Events Surveillance System (PHESS). PHESS is a centralised surveillance database containing data on all notifiable diseases in Victoria since 1991 [6]. The notification of active tuberculosis cases is mandatory in Victoria under the Public Health and Wellbeing legislation [6]. PHESS has standardised data collection templates to ensure consistency. Nurse consultants record patient demographic, clinical data and TB contacts in PHESS.

Data on the estimated resident population for all local government areas were obtained from the Australian Bureau of Statistics (ABS). The estimated resident population is the official figure of Australia's population based on the concept of "usual residence" and refers to all people, regardless of nationality or citizenship, who usually live in Australia, except foreign diplomatic personnel and their families [8].

Study design

We conducted a respective cohort study. Patients notified to the Australian department of health with active TB from 1995 to 2019 were identifie[illegible]We analysed the data of these patients from the time they first developed TB symptoms until they completed TB treatment. Our study adhered to the Strengthening The Reporting of Observational Studies in Epidemiology (STROBE) guidelines (see S7 Table).

Study setting

The fieldwork for this study was conducted in Victoria by the VTP staff. Funded by the Victorian Government Department of Health, VTP is a centralised program located in metropolitan Victoria and works in partnership with hospitals and clinics in managing tuberculosis. All tuberculosis patients in Victoria are supervised by VTP nurse consultants [9,10].

Study Population

The population for this study included all people of all ages who had been diagnosed with tuberculosis and notified to the department of health.

Inclusion criteria.

Patients were included in the study if they met the following inclusion criteria:

1. Diagnosed with TB in Victoria and notified to the Victorian department of health from 1 January 1995 to 31 December 2019. TB cases were defined in accordance with a standard national case definition based on either laboratory definitive evidence requiring isolation of Mycobacterium tuberculosis complex by culture or nucleic acid testing or clinical diagnosis accompanied by treatment [11].
2. Having received tuberculosis treatment in Victoria.

Exclusion criteria.

Patients were excluded from the study if they:

1. were notified before 1995 or after 2019
2. Lacking residential addresses

Variab[illegible]

The dependent variables were the treatment delays and the treatment outcomes (study outcomes). Treatment outcomes included completed treatment, lost to follow up, died of TB, died of other causes during treatment for TB, and transferred interstate or overseas. For the treatment delays, we adapted the definitions outlined by Van Wyk et al. [12], which proposed that: ‘Patient treatment delay’ is the period (in the number of days) between the onset of any self-reported TB symptoms and the first visit to a health care facility. ‘Health system delay’ is the period (in the number of days) between the first health care facility visit and initiation of TB treatment. ‘Diagnostic delay’ is defined as the period (in the number of days) between the onset of any self-reported TB-related symptoms and the time a chest x-ray was performed. ‘Treatment initiation delay’ was the period between a positive specimen (TB confirmed) and treatment initiation.

We also extracted the following independent variables from PHESS, (1) demographic data: age (age groups in years), sex (male or female), country of birth (name of the country), Aboriginal and Torres Strait Islander status (Aboriginal and/or Torres Strait Islander or not Aboriginal and/or Torres Strait Islander), local government areas (local government area), self-reported residency status (Australian-born, permanent resident, refugee/humanitarian, visitor, overseas student, other and unknown status), and for overseas-born cases, year of arrival in Australia (year). (2) Clinical characteristics: year of tuberculosis notification (year), the manifestation of tuberculosis (pulmonary, extrapulmonary or both), chest X-ray results (abnormal, cavitation or normal), laboratory results (smear, culture, or gene expert), and treatment outcome (died of TB, died of other causes during treatment for TB, complete[illegible]reatment, lost to follow up, and transferred interstate or overseas).

Ethical considerations

Approval from a Human Research Ethics Committee for this study was not required as the data were collected for the purposes of public health action, as defined in the Public Health and Wellbeing Act 2008 and were considered as being for quality assurance and auditing purposes. Patients were informed of the purpose of data collection and consented to their data being used for tuberculosis surveillance and medical research at the time of collection. All data were fully anonymised during the data extraction process. For example, participants' names, phone numbers, addresses, and birth dates were removed. Patient identification numbers, postcodes, and gender were coded. Age was changed to age group. In the publications that come from this study, patients will remain anonymous.

Data analysis

Data cleaning and analyses were conducted using STATA version 14.

Managing missing data

We used a listwise deletion method when missing data contained residential addresses (participants were allocated neither to regional nor metropolitan areas) because our exposure of interest was regionality. When missing data did not have the key variable (i.e., residential address), we utilised the pairwise deletion approach, which allowed us to retain data and reduce the possibility of selection bias.

Analysis

Descriptive and multivariable analyses were performed. Incidence rates were calculated using the mid-year estimated resident population. Pearson's x^2 test was used to test the association between categorical variables. A two-tailed p-value of <0.05 was considered statistically significant. In logistic regression, we compared complete treatment with death, irrespective of the cause, lost to follow-up and transferred interstate or overseas. Died of TB was compared with completed treatment, lost to follow-up, died of other causes during treatment for TB, and transferred interstate or overseas.

We included all independent variables in all multivariable analyses because we believed they could all affect the outcomes. Our variable of interest was regionality. The proportional-hazards assumption was assessed using Kaplan-Meier survival curves by including time-dependent covariates in the model and with Schoenfeld residuals. In cases where proportionality assumptions were not met, analyses were stratified. Kaplan-Meier survival curves were used to show various delays in presentation, diagnosis, and treatment between regional and metropolitan cohorts, and Cox proportional hazard analyses were performed to assess these delays. Patient follow-up was censored, 1. at the first visit to a health care facility (Patient treatment delay), 2. at the initiation of TB treatment (Health system delay), 3. at the time a chest x-ray was performed (Diagnostic delay), 4. at the treatment initiation (Treatment initiation delay).

Because of limited previous data, treatment outcomes were analysed using data from 2005 to 2019, and for the analysis of treatment delays, we used data from 2012 to 2019.

Results

A total of 8,819 TB cases were notified to the Victorian Government Department of Health between 1995 and 2019. Among the 8,819 cases, 611 (7%) were recorded in regional areas, 8,163 (93%) in metropolitan areas of Victoria and 45 (1%) had neither regional nor metropolitan residential addresses (see Fig 1). Forty-five cases with no residential addresses were excluded from the study as they were classified as neither regional nor metropolitan. Of the 611 people in regional areas, 343 (56%) were male, 401 (66%) were overseas-born, and for 10 cases (2%), there was no country of birth recorded. Among the 8,163 TB cases in metropolitan areas, 4,316 (53%) were male, 8 (0.1%) had no gender reported and 7,375 (90%) were overseas-born.

Fig 1. The flow of patients through the study

Data recorded before 2005 had missing treatment outcomes for many cases and were therefore excluded from the analysis of treatment outcomes (Fig 1). The overall treatment completion rates were similar among the regional and metropolitan cohorts: 85% and 90%, respectively.

Table 1 describes the characteristics of notified TB cases in Victoria from 1995 to 2019 by location of residence and birth. The proportion of overseas-born cases in the regional cohort was 66% compared to 90%, in metropolitan areas. The 25 to 34 age group had the largest proportion of cases in both regional and metropolitan areas. In this age bracket, there were 27% of overseas-born and 8% of Australian-born cases in regional settings, and 31% of overseas-born and 11% of Australian born cases in metropolitan areas. The proportions of multidrug-resistant TB (MDR-TB) cases among the regional and metropolitan patients were similar. The four MDR-TB cases reported in the regional cohort all occurred in people born

overseas. In the metropolitan cohort, the proportion of MDR-TB cases was the same (1%) amongst overseas and Australian-born persons. Extensive drug-resistant TB (0.03%) and genotypic rifampicin-resistant TB (0.04%) were only reported in the metropolitan area among overseas-born cases.

Table 1. Characteristics of the notified cases of Tuberculosis in Victoria from 1995 to 2019.

Variable		Regional (n = 601)				Metropolitan (n = 8,163)			
		Overseas-born (n = 401)		Australian-born (n = 200)		Overseas-born (n = 7,375)		Australian born (n = 788)	
		Total	Proportion %	Total	Proportion %	Total	Proportion %	Total	Proportion %
Gender	Male	215	54	123	62	3,869	52	447	57
	Female	186	46	77	39	3,500	47	339	43
	Unknown	0	0	0	0	6	0	2	0
Age group	Under 5	3	1	11	6	31	0	115	15
	5-14	12	3	7	4	122	2	79	10
	15-24	49	12	14	7	1,303	18	129	16
	25-34	110	27	15	8	2,272	31	87	11
	35-44	60	15	10	5	1,188	16	60	8
	45-54	49	12	15	8	715	10	67	9
	55-64	33	8	31	16	562	8	56	7
	65 and above	85	21	97	49	1,181	16	195	25
	Unknown	0	0	0	0	1	0	0	0
Manifestation	Pulmonary	193	48	131	66	2,864	39	459	58
	Pulmonary Plus other sites	42	10	18	9	936	13	117	15
	Extra Pulmonary	162	40	49	25	3,396	46	206	26
	Unknown	4	1	2	1	179	2	6	1
Susceptibility	Fully sensitive	233	58	117	59	4,376	59	402	51
	Multidrug - resistant tuberculosis	4	1	0	0	88	1	9	1
	Other resistance	12	3	2	1	379	5	29	4
	Extensively drug-resistant tuberculosis	0	0	0	0	2	0	0	0
	Genotypic Rifampicin resistant tuberculosis	0	0	0	0	3	0	0	0
	Unknown	152	38	81	41	2,527	34	348	44

Treat outcome (from 2005 to 2019	Completed treatment	238	86	88	82	4,491	90	442	92
	Lost to follow-up	5	2	4	4	106	2	8	2
	Died from other cause	12	4	12	11	128	3	22	5
	Died of tuberculosis	4	1	3	3	58	1	5	1
	Still on treatment at the time of data extraction	1	0	0	0	1	0	0	0
	Transferred interstate or overseas	18	6	0	0	222	4	1	0
	Unknown	0	0	0	0	1	0	0	0

Note: Treatment outcome regional, n = 385 and Metro, n = 5,485.

The number of tuberculosis cases in regional Victoria has fluctuated over time, with 129 notified from 1995 to 1999, 97 from 2000 to 2004, and 155 from 2015 to 2019. Table 2 compares the TB incidence rate between regional and metropolitan areas. From 1995 to 1999, there were 129 cases with a mean incidence rate of 2.0, 95% CI 1.3-2.7 per 100,000 population in regional Victoria, while in the metropolitan there were 1,271 cases with a mean incidence rate of 7.7, 95% CI 6.9-8.4. The TB incidence in regional and metropolitan areas is fluctuating; in the 2015 to 2019 period, the mean incidence for the regional cohort was 2.1, 95% CI 1.5-2.7 and 7.9, 95% CI 7.3-8.4 in the metropolitan.

Table 2. Tuberculosis incidence rate per 100,000 population in regional Victoria and metropolitan Victoria from 1995 to 2019.

Years	Regional areas		Metropolitan areas	
	Tuberculosis cases	Mean incidence rate per 100,000 population (95% CI)	Tuberculosis cases	Mean incidence rate per 100,000 population (95% CI)
1995-1999	129	2.0 (1.3-2.7)	1,271	7.7 (6.9-8.4)
2000-2004	97	1.5 (1.0-1.9)	1,407	8.0 (7.6-8.4)
2005-2009	98	1.4 (1.2-1.6)	1,742	9.1 (8.9-9.4)
2010-2014	132	1.8 (1.4-2.1)	1,841	8.7 (7.9-9.5)
2015-2019	155	2.1 (1.5-2.7)	1,902	7.9 (7.3-8.4)

TB cases among overseas-born people aged 20 to 49 years have rapidly increased since 2004 (Fig 2). Conversely, in the Australian-born population aged 20 to 49 years, cases have remained stable since 1995. The number of TB cases among Australian-born people aged ≥50 years has decreased from 1995 and slightly upturned since 2014.

Fig 2. Tuberculosis cases in regional Victoria by year of notification, age, and country of birth from 1995 to 2019.

A total of 386 overseas-born cases had their year of arrival in Australia recorded. Half of the people (197; 51%) developed TB disease within five years of arrival in Australia. Out of these 197 cases, 58 (29%) were permanent residents, 43 (22%) were refugees, 19 (10%) were visitors, 18 (9%) were overseas students, and 59 (30%) had unknown residential status. Among 19 overseas students, 18 developed TB within five years and one between six

and 11 years of arrival. Of the 46 refugees recorded in the study, 43 were diagnosed with TB within five years of arrival. The risk of developing TB remained for many years after people arrived in Australia; 10 (3%) people were diagnosed with TB after 53 years of arrival (see, Fig 3).

Fig 3. Notified cases of tuberculosis in the overseas-born people in regional Victoria from 1995-2019 by the number of years since arrival in Australia.

In 2016, the TB incidence rate for the Australian-born population was 0.5 per 100,000 people and 12.0 per 100,000 people born overseas. Thirteen frequently reported countries of birth for the overseas-born cases from 1999 to 2019 are shown in Fig 4. People born in these 13 countries make up 62% of the overseas-born cases during the study period. India had the highest number of notified cases, 58 (14%), followed by the Philippines, 48 (12%). People born in India and the Philippines accounted for 26% of all the TB cases in regional areas.

Fig 4. Notified cases of tuberculosis in regional Victoria for overseas-born people from 1999 to 2019, by country of birth.

Among 5,870 cases with known treatment outcomes, 5,259 (90%) completed treatment, 124 (2%) were lost to follow-up, 174 (3%) died of another cause while on TB therapy, 70 (1%) died of TB, 2 (0.03%) were still on treatment at the time of data extraction, and 241 (4%) were transferred either interstate or overseas. Table 3 shows univariable and multivariable analyses of predictors of treatment completion and dying of TB. Living in a regional area was associated with lower odds of treatment completion on univariable analysis (OR = 0.6, 95% CI 0.5-0.8, P = 0.002). After adjusting for the effect of age, sex, drug

susceptibility and country of birth in the model, living in a regional area remained significantly associated with lower treatment completion (Adjusted OR [AOR] = 0.7, 95% CI 0.5-0.9, P = 0.019). On multivariable analysis, older age and male sex were also predictors of lower treatment completion.

Living in a regional area did not significantly increase the odds of dying from TB on univariable analysis (OR = 1.6, 95% CI 0.7-3.5, P = 0.244). In a model that included all the five variables in the multivariable analysis, regionality was not associated with dying of TB (AOR = 1.8, 95% CI 0.7-4.2, P = 0.198). Older age was significantly associated with dying of TB.

Table 3. Univariable and multivariable analysis of predictors of treatment completion and dying of tuberculosis from 2005 to 2019.

Variable	Treatment completion				Died of tuberculosis			
	Univariable analysis		Multivariable analysis		Univariable analysis		Multivariable analysis	
	OR (95%)	P valve	AOR (95%)	P valve	OR (95%)	P valve	AOR (95%)	P valve
Regional (reference group: metropolitan cases)	0.6 (0.5-0.8)	0.002	0.7 (0.5-0.9)	0.019	1.6 (0.7-3.5)	0.244	1.8 (0.7-4.2)	0.198
Age ≥ 65 years (reference group: age < 65 years)	0.2 (0.2-0.3)	<0.0001	0.2 (0.2-0.3)	<0.0001	12.6 (7.6-20.9)	<0.0001	9.9 (5.8-16.9)	<0.0001
Male sex (reference group: female sex)	0.6 (0.5-0.7)	<0.0001	0.7 (0.6-0.8)	<0.0001	1.8 (1.1-3.0)	0.020	1.5 (0.9-2.6)	0.131
Drug resistant tuberculosis (reference group: fully sensitive tuberculosis)	0.8 (0.6-1.1)	0.228	0.8 (0.6-1.1)	0.128	1.7 (0.8-3.5)	0.179	1.9 (0.9-4.1)	0.104
Overseas-born (reference group: Australian-born	0.9 (0.7-1.2)	0.415	0.7 (0.5-1.1)	0.094	0.9 (0.4-1.8)	0.682	1.8 (0.7-4.9)	0.238

Notes: AOR is an adjusted odds ratio.

The effect of regionality in multivariable Cox proportional hazard model analyses is shown in Table 4. The model included age, sex, place of birth, place of residence, and drug susceptibility. Data prior to 2012 were incomplete in relation to TB symptom onset, chest X-

ray findings and laboratory investigations and were therefore unable to be included in the analyses of delay in TB diagnosis and treatment. A total of 241 cases in regional and 2,997 in metropolitan areas were included.

Patient and health system delays were similar in regional and metropolitan areas for cases with pulmonary involvement. In regional areas, people with pulmonary involvement underwent chest x-ray (diagnostic delay) slightly sooner than those notified in metropolitan areas (median six days versus nine days, AHR = 1.2, 95% CI 1.0-1.5, P = 0.047). Conversely, healthcare system delay was non-significantly longer in regional than in metropolitan patients (median 64 days versus 54 days, AHR = 0.8, 95% CI 0.6-1.0, P = 0.094).

Table 4. Adjusted relationship between regionality in Cox regression models of delays in the cascade of care among tuberculosis patients in Victoria from 2012 to 2019.

			Effect of regional in Cox regression analysis			
Time period outcome	Regional area Median (interquartile range) days	Metropolitan area Median (interquartile range) days	Number observed in regional	Number observed in metropolitan	Adjusted Hazard ratio (95% CI)	P-value
Patient with pulmonary involvement						
Patient delay	21 (1-76)	24 (1-67)	113	1,293	0.9 (0.8-1.1)	0.389
Health system delay	21.5(7-44)	25 (7-65)	146	1,691	1.2 (1.0-1.4)	0.102
Diagnostic delay: Presentation to First chest-x-ray	6 (0-27)	9 (0-41)	122	1,343	1.2 (1.0-1.5)	0.047

Treatment delay: First chest X-ray to start of tuberculosis treatment	8 (3-23)	9 (3-29)	131	1,565	1.0 (0.9-1.3)	0.614
Extrapulmonary patients						
Patient delay	11.5 (0-68)	23 (0-75)	62	907	1.0 (0.7-1.2)	0.756
Health system delay	64 (26-137)	54 (21-112)	78	1,107	0.8 (0.6-1.0)	0.094
Diagnostic delay: Presentation to First chest x-ray	26 (5-92)	30 (5-72)	47	798	0.9 (0.7-1.2)	0.393
All patients						
Treatment delay 2: specimen test to treatment initiation	7 (1-19)	5 (1-17)	151	1,996	1.0 (0.8-1.2)	0.747

The Kaplan-Meier curves for patient delay are shown in Fig 5.

Fig 5. Kaplan-Meier curves in tuberculosis patients in Victoria, Australia from 2012 to 2019.

Discussion

We report the trends and treatment outcomes of notified TB cases in regional areas of Victoria, Australia, from 1995 to 2019. The incidence of TB is low in regional areas, and this is consistent with the findings from studies reported in the United States of America (USA)

and the United Kingdom. For example, a study in Appalachia, USA, reported that in 2005 the rate of tuberculosis in regional Appalachia was 2.1 compared to 2.7 per 100,000 population in metropolitan areas [13]. A similar study conducted in England and Wales from 2001 to 2003 found that the rate of TB in metropolitan areas was 6.3-fold higher compared to regional areas [14].

We observed that there had been a slight increase in TB incidence in regional Victoria from 1995 to 2019. The increase in TB incidence in regional Victoria is not surprising considering the increase in people born in high TB incidence countries [2]. In the regional cohort, the proportion of TB among the overseas-born population was twice that of the Australia-born people. There is an increase in TB cases aged between 20 and 49 years among the overseas-born in the regional cohort. These results suggest there may be a public health benefit in increasing latent TB detection and treatment in regional areas, targeting 20-49-year-old overseas-born people, and offering TB preventative therapy to those found with latent TB.

In regional Victoria, TB resistance was more common in overseas-born cases, consistent with other Australian studies [6,15]. We analysed the time from arrival in Australia to TB diagnosis for overseas-born cases in the regional cohort. More than half of the overseas-born cases were notified within five years of arrival in Australia. The high TB notification within the first five years of arrival may be attributed to the latent TB reactivation [16]. It is worth noting that the majority of the refugees were diagnosed within five years of arrival. This may be a result of more intensive screening soon after arrival, including testing for LTBI in asylum seekers but not migrants more generally. Refugees may also return overseas less frequently than other migrants and be less likely to be reinfected.

In this study, health-seeking behaviour and treatment outcomes were similar between the regional and metropolitan settings. These results suggest that existing programs are functioning well, although the possible trend toward health service delays requires further monitoring and reviewing opportunities for programmatic strengthening. In addition, people aged over 64 years are at significantly greater risk of dying from TB and, in appreciation of this risk, more intensive care may be required.

Due to a paucity of research in regional areas of countries with a low incidence of TB, we cannot make a direct comparison between our study and other published literature. Putting aside the comparison between regional and metropolitan data, our results in relation to delays in TB diagnosis and treatment are consistent with other Australian studies as well as systematic reviews [10,17,18]. For example, Bello et al., [17] performed a systematic review of 198 studies. They reported a median duration of patient delay of 28 days and a health system delay of 18 days compared to 21 days for each of these categories for the regional patients in our study. Of interest, extrapulmonary tuberculosis in our cohort had a much longer health system delay, averaging 64 days for regional patients.

Limitations

Strengths of this study include the use of a comprehensive central database that includes important demographic, clinical and laboratory data, allowing for the incorporation of other factors outlined in this manuscript and a long study period of 25 years. However, we acknowledge that data for the entire study period are not available for all data fields (e.g., treatment outcomes, health system delays), limiting trend analysis. Some of the data in our study, such as dates of symptom onset and healthcare presentation, were collected retrospectively from patients and thus may contain inaccuracies relating to recall bias. Data

on some factors that could have influenced the treatment delay and outcomes, such as educational level was limited.

Conclusion

Tuberculosis in regional Victoria is more common among the overseas-born population, and patients with extrapulmonary TB in regional areas have non-significant minor delays in treatment commencement. Increasing migration from high incidence TB countries to regional settings in Australia requires an ongoing review of available and accessible health services to limit delays in timely diagnosis and treatment. Increasing access to LTBI management and enhanced diagnostic pathways in regional areas may assist in reducing the burden and impact of TB in the future.

Author Contributions

Nompilo Moyo: Conceptualization, Formal analysis, Methodology, Project administration, Writing – original draft.

Tay EL: Data curation, Writing – review & editing.

Trauer JM, Burke L, Boyd SC, Singh KP, Jackson J, Commons RJ: Writing – review & editing.

Denholm JT: Conceptualization, Supervision, Writing – review & editing.

Acknowledgements

The authors would like to thank the clinical nurse consultants for collecting and entering data into the database.

References

1. Australian Bureau of Statistics. National, state and territory population. [Internet]. Australian Bureau of Statistics.; 2020 [cited 2020 Nov 30]. Available from: https://www.abs.gov.au/statistics/people/population/national-state-and-territory-population/latest-release

2. Australian Bureau of Statistics. 2016 Census Community Profiles. [Internet]. Australian Bureau of Statistics.; 2017 [cited 2020 Nov 30]. Available from: https://quickstats.censusdata.abs.gov.au/census_services/getproduct/census/2016/communityprofile/2RVIC?opendocument

3. Department of Home Affairs, Australia. Regional migration. [Internet]. Department of Home Affairs, Australia.; 2020 [cited 2020 Nov 30]. Available from: https://immi.homeaffairs.gov.au/visas/working-in-australia/regional-migration

4. Trauer JM, Williams B, Laemmle-Ruff I, Horyniak D, Caplice LVS, McBryde ES, et al. Tuberculosis in migrants to Australia: Outcomes of a national screening program. Lancet Reg Health West Pac. 2021;10:100135–100135.

5. McBryde ES, Denholm JT. Risk of active tuberculosis in immigrants: effects of age, region of origin and time since arrival in a low-exposure setting. Med J Aust. 2012;197(8):458–61.

6. Bright, A., Denholm, J., Coulter, C., Waring, J., & Stapledon, R. Tuberculosis notifications in Australia, 2015-2018. Commun Dis Intell. 2020;44:1–39.

7. World Health Organisation. Strengthening TB surveillance. [Internet]. 2022 [cited 2022 Jun 26]. Available from: https://www.who.int/westernpacific/activities/strengthening-tb-surveillance

8. Australian Bureau of Statistics. 3107.0.55.006 - Information Paper: Population Concepts, 2008 [Internet]. Australian Bureau of Statistics.; 2008 [cited 2021 Jan 12]. Available from: https://www.abs.gov.au/AUSSTATS/abs@.nsf/Lookup/3107.0.55.006Glossary12008?OpenDocument

9. Moyo N, Tay EL, Nolan A, Graham HR, Graham SM, Denholm JT. TB contact tracing for young children: an Australian cascade of care review. Public Health Action. 2021;11(2):91–6.

10. Dale KD, Tay EL, Trauer JM, Trevan PG, Denholm JT. Comparing tuberculosis management under public and private healthcare providers: Victoria, Australia, 2002-2015. BMC Infect Dis. 2017;17(1):324–324.

11. Australian Government Department of Health. Tuberculosis case definition [Internet]. Australian Government Department of Health; 2010 [cited 2022 Apr 12]. Available from: https://www1.health.gov.au/internet/main/publishing.nsf/Content/cda-surveil-nndss-casedefs-cd_tb.htm

12. VAN WYK SS, ENARSON DA, BEYERS N, LOMBARD C, HESSELING AC. Consulting private health care providers aggravates treatment delay in urban South African tuberculosis patients. Int J Tuberc Lung Dis. 2011;15(8):1069–76.

13. Wallace RM, Armstrong LR, Pratt RH, Kammerer JS, Iademarco MF. Trends in Tuberculosis Reported From the Appalachian Region: United States, 1993-2005. J Rural Health. 2008;24(3):236–43.

14. ABUBAKAR I, CROFTS JP, GELB D, STORY A, ANDREWS N, WATSON JM. Investigating urban–rural disparities in tuberculosis treatment outcome in England and Wales. Epidemiol Infect. 2007;136(1):122–7.

15. Toms C, Stapledon R, Waring J, Douglas P. Tuberculosis notifications in Australia, 2012 and 2013. Commun Dis Intell. 2015;39(2):E217–35.

16. Dale KD, Trauer JM, Dodd PJ, Houben RMGJ, Denholm JT. Estimating Long-term Tuberculosis Reactivation Rates in Australian Migrants. Clin Infect Dis. 2020;70(10):2111–8.

17. Bello S, Afolabi RF, Ajayi DT, Sharma T, Owoeye DO, Oduyoye O, et al. Empirical evidence of delays in diagnosis and treatment of pulmonary tuberculosis: systematic review and meta-regression analysis. BMC Public Health. 2019;19(1):820–820.

18. Williams E, Cheng AC, Lane GP, Guy SD. Delays in presentation and diagnosis of pulmonary tuberculosis: a retrospective study of a tertiary health service in Western Melbourne, 2011–2014. Intern Med J. 2018;48(2):184–93.

Figure 1

Click here to access/download;Figure;Plos one Fig 1 .tif

Fig 1. The flow of patients through the study

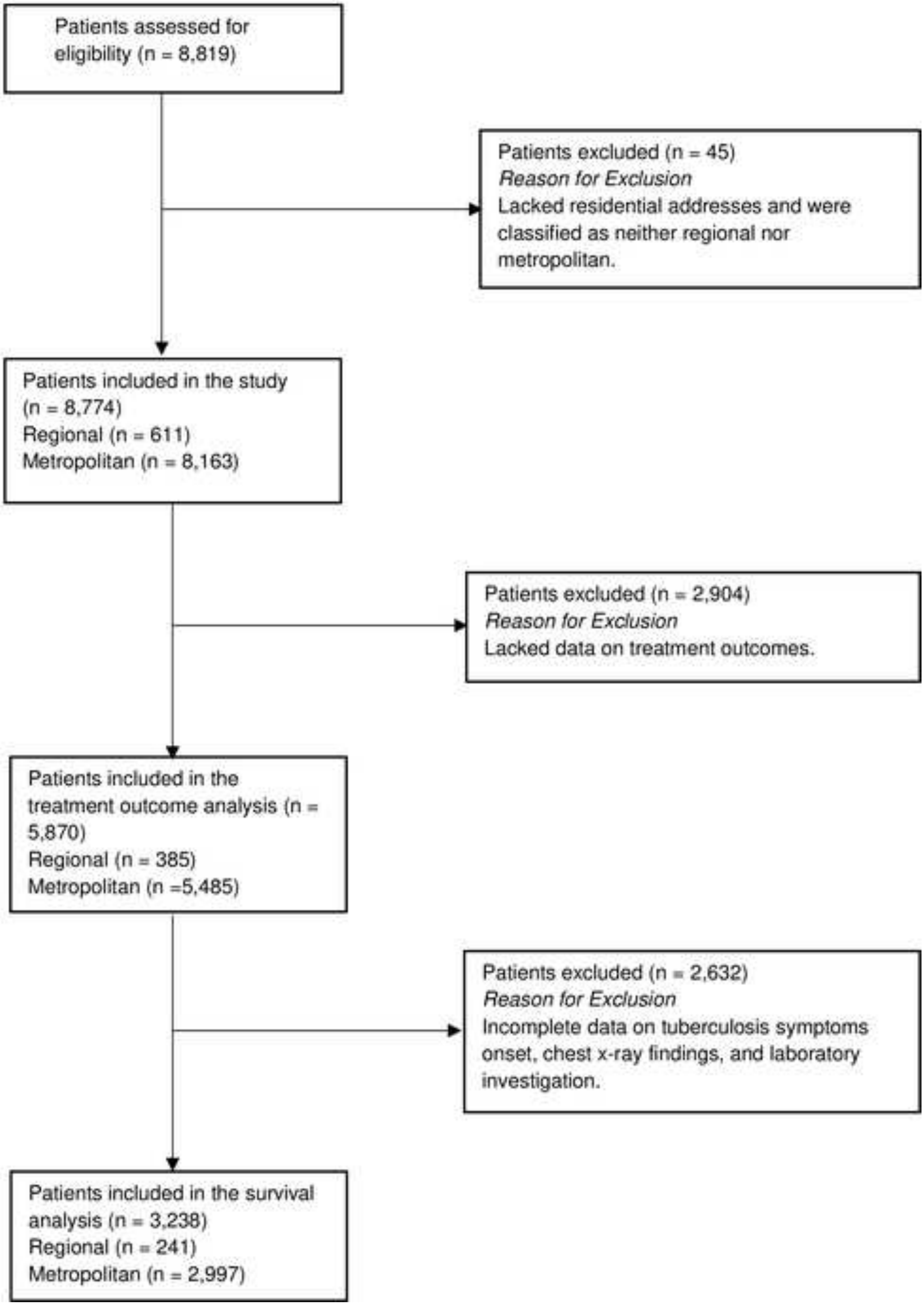

Figure 2

Click here to access/download;Figure;Plos one Fig 2 .tif

Fig 2. Tuberculosis cases in regional Victoria by years of notification, age and country of birth from 1995 to 2019.

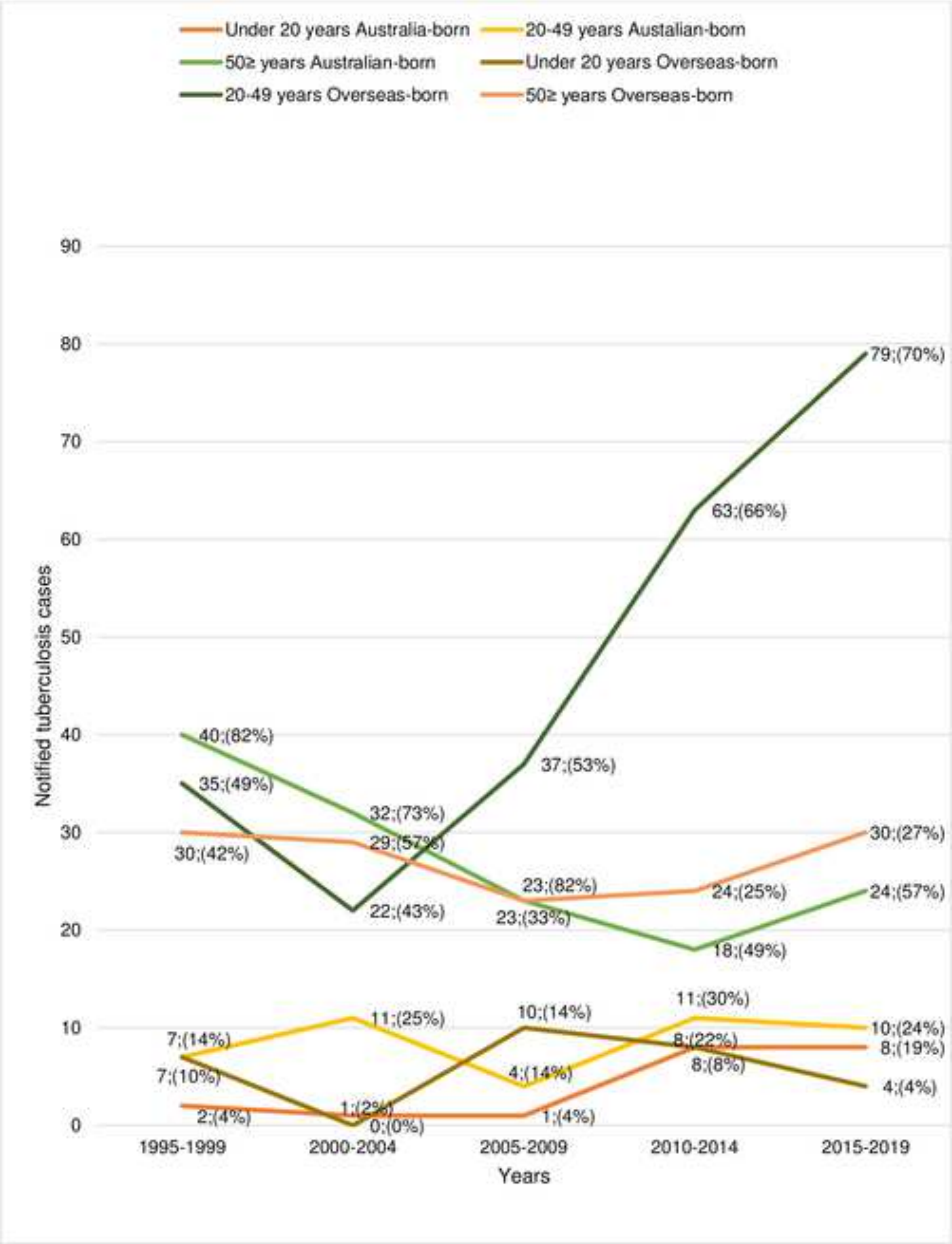

 Click here to access/download;Figure;Plos one Fig 3.tif

Fig 3. Notified cases of tuberculosis in overseas-born people in regional Victoria from 1995-2019 by the number of years since arrival in Australia.

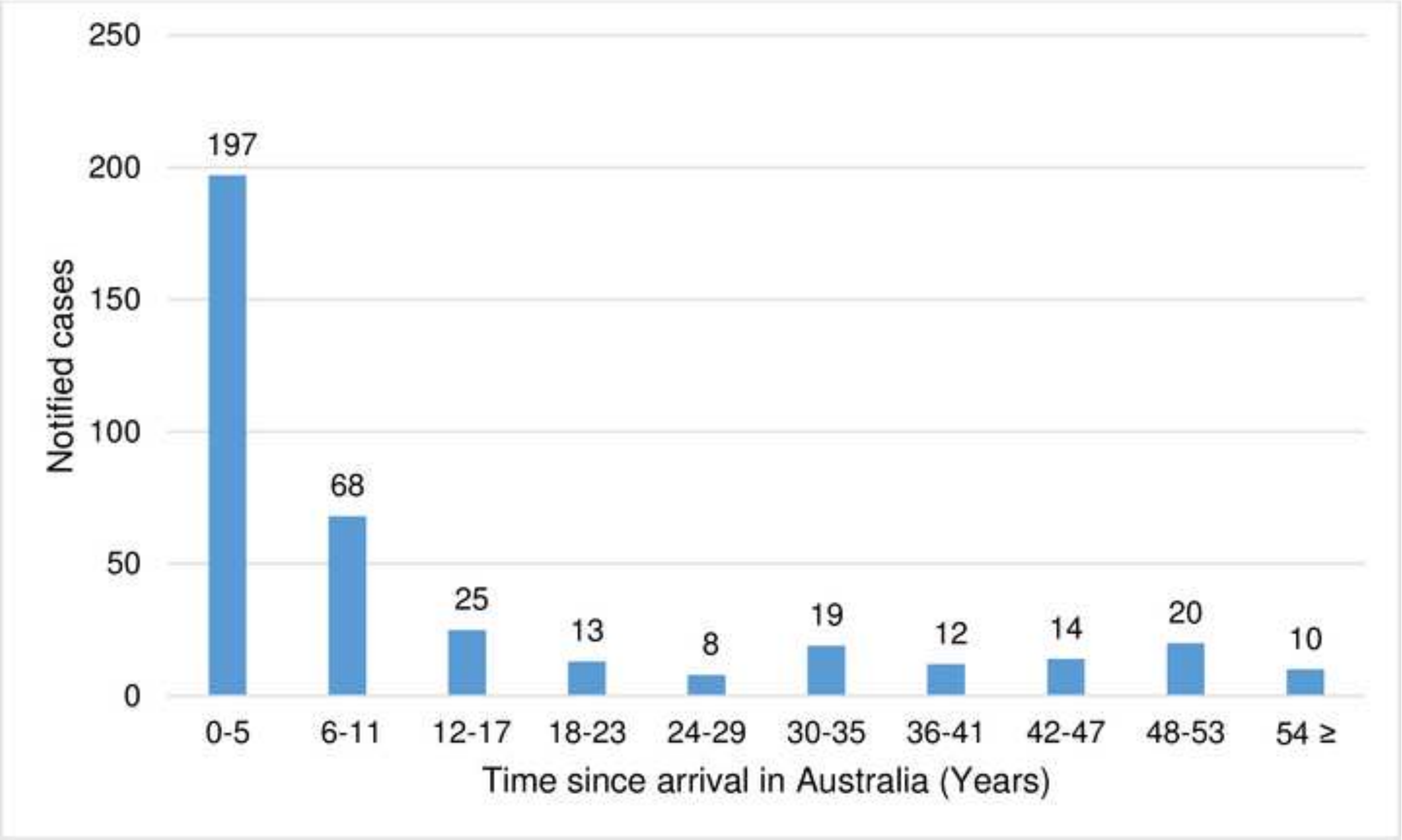

Figure 4

Click here to access/download;Figure;Plos one Fig 4 .tif

Fig 4. Notified cases of tuberculosis in regional Victoria for overseas-born people from 1999 to 2019, by country of birth.

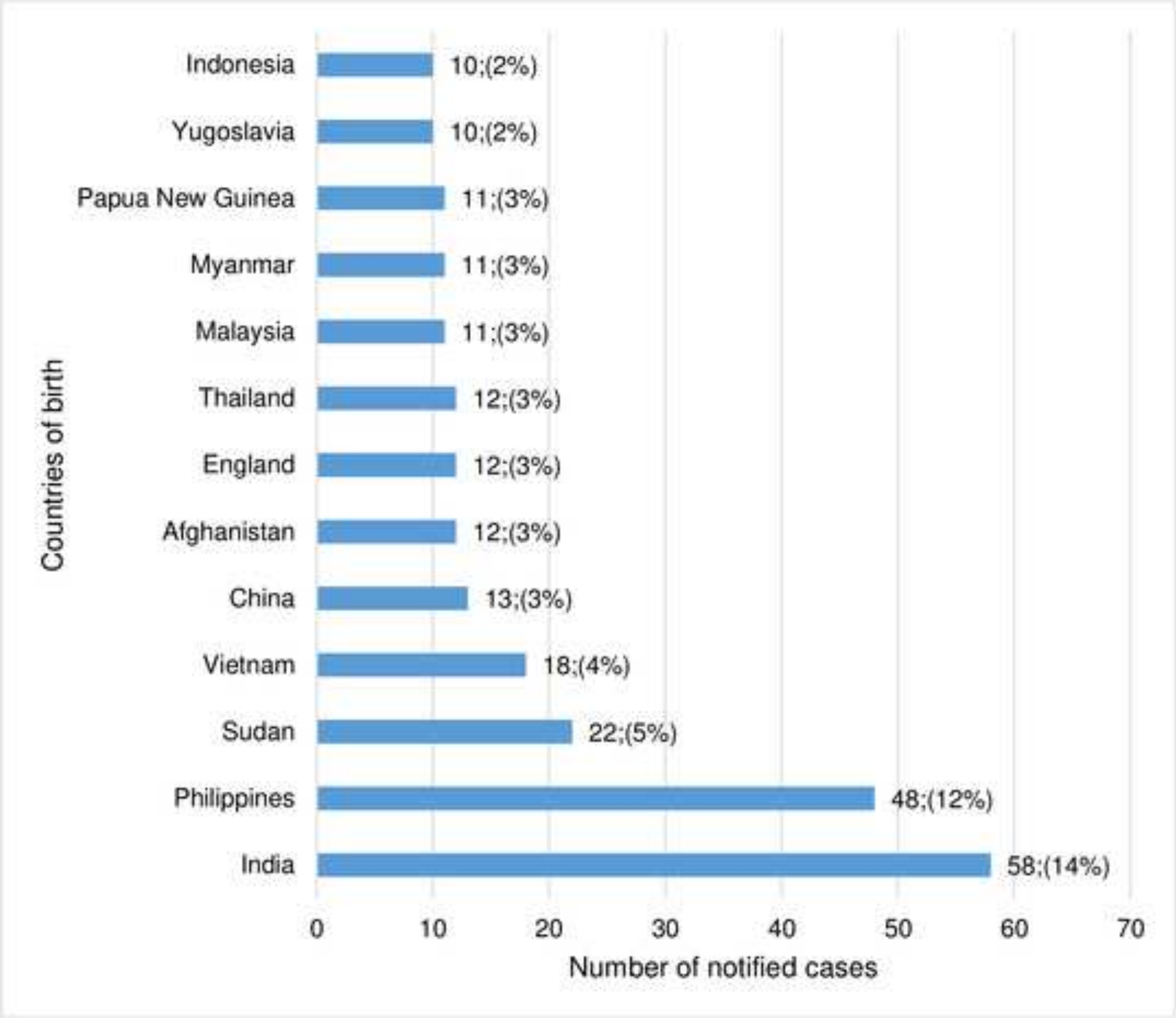

Notes: Yugoslavia no longer exist.

Click here to access/download;Figure;Plos one Fig 5 .tif

Fig 5. Kaplan-Meier curves in tuberculosis patients in Victoria, Australia, from 2012 to 2019. Black = regional and grey = metropolitan.

Diagnostic delay to first CXR for patients with pulmonary involvement

Health system delay for patients with extrapulmonary tuberculosis

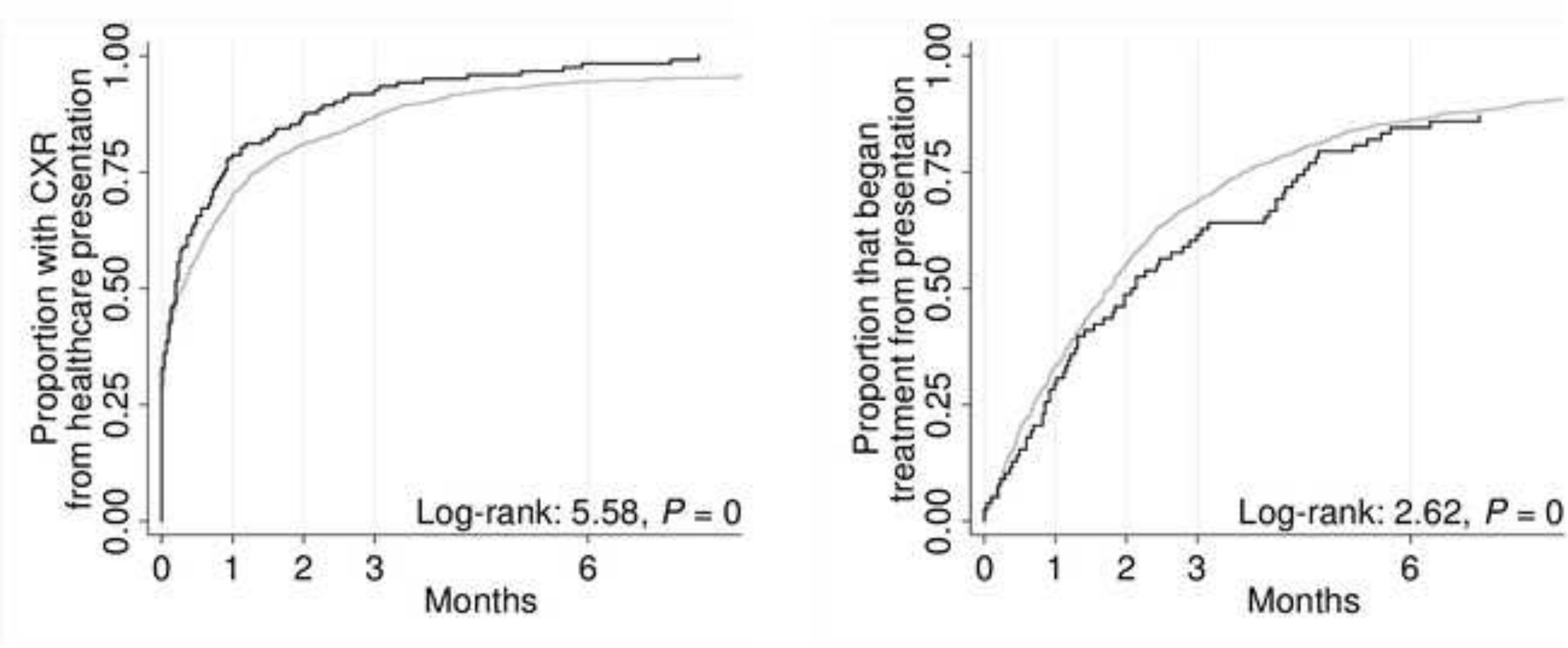

Click here to access/download
Supporting Information
TB notifications in regional Victoria PLOS ONE SI_1.docx

Revised Manuscript with Track Changes

Tuberculosis notifications in regional Victoria, Australia: implications for public health care in a low incidence setting

Moyo N[1, 2*], Tay EL[3], Trauer JM[1, 4] Burke L[1], Jackson J[5, 6], Commons RJ[7, 8], Boyd SC[9], Singh KP[10], Denholm JT[1, 10]

[1] Victorian Tuberculosis Program, Melbourne Health, Melbourne, Victoria, Australia

[2] La Trobe University, School of Nursing and Midwifery, Melbourne, Victoria, Australia

[3] Communicable Diseases Epidemiology and Surveillance Unit, Health Protection Branch, Public Health Division, Department of Health, Victoria, Australia

[4] Monash University, School of Public Health and Preventive Medicine, Melbourne, Victoria, Australia

[5] Department of Medicine, Albury-Wodonga Health, Wodonga, Victoria, Australia

[6] Faculty of Medicine, University of New South Wales Rural Clinical School, Albury Campus, New South Wales, Australia

[7] Internal Medicine Services, Ballarat Health Services, Ballarat, Victoria, Australia

[8] Global and Tropical Health Division, Menzies School of Health Research and Charles Darwin University, Darwin, Northern Territory, Australia

[9] Royal Brisbane and Women's Hospital, Brisbane, South Australia, Australia

[10] Department of Infectious Diseases, Doherty Institute for Infection and Immunity, University of Melbourne, Parkville, Victoria, Australia

∗ nompiloo@yahoo.com

Abstract

Background: Regionality is often a significant factor in tuberculosis (TB) management and outcomes worldwide. A wide range of context-specific factors may influence these differences and change over time. We compared TB treatment in regional and metropolitan areas, considering demographic and temporal trends affecting TB diagnosis and outcomes. Methods: Retrospective analyses of data for patients notified with TB in Victoria, Australia, were conducted. The study outcomes were treatment delays and treatment outcomes. Multivariable Cox proportional hazard model analyses were performed to investigate the effect of regionality in the management of TB. Six hundred and eleven (7%) TB patients were notified in regional and 8,163 (93%) in metropolitan areas between 1995 and 2019. Of the 611 cases in the regional cohort, 401 (66%) were overseas-born. Fifty-one percent of the overseas-born patients in regional Victoria developed TB disease within five years of arrival in Australia. Four cases of multidrug-resistant tuberculosis were reported in regional areas, compared to 97 cases in metropolitan areas. A total of 3,238 patients notified from 2012 to 2019 were included in the survival analysis. Patient follow-up was censored at the first visit to the health care facility (Patient treatment delay) and at the initiation of TB treatment (Health system delay). Patient, health system, and treatment delays were similar in regional and metropolitan areas for cases with pulmonary involvement. Cases with extrapulmonary TB in regional areas have a non-significantly longer healthcare system delay than patients in metropolitan (median 64 days versus 54 days, AHR = 0.8, 95% CI 0.6-1.0, P = 0.094).

Conclusion: Tuberculosis in regional Victoria is common among the overseas-born population, and patients with extrapulmonary TB in regional areas experienced a non-significant minor delay in treatment commencement with no apparent detriment to

treatment outcomes. Improving access to LTBI management in regional areas may reduce the burden of TB.

Keywords: regional, metropolitan, tuberculosis, treatment completion, delayed diagnosis

Word count: 3,715 words, excluding tables and references.

Background

Victoria is the second most highly populated state in Australia, with 6.69 million residents as of March 2020 [1]. It has the highest population growth rate (1.8%), with net overseas migration functioning as the primary contributor to population growth in Victoria [1].

Victoria is divided into two distinct socio-geographic areas, metropolitan and regional. Metropolitan is defined as the 31 local government areas of the city of Melbourne, while the 48 local government areas outside of the city are defined as regional. Based on 2016 census data, 4,485,211 of Victoria's total population of 5,926,624 lived within Melbourne, with less than 25% of the population living regionally [2]. This equates to an average population density of 500 people per square kilometre in metropolitan Victoria compared to an average of 6 people per square kilometre in regional Victoria.

There are some differences in the provision of health care services between Victoria's metropolitan and regional areas. Unlike patients in most metropolitan areas, patients in regional Victoria may reside a considerable distance from hospitals. Most regional hospitals do not have negative pressure rooms for isolating TB patients during their infectious stage; therefore, infectious patients need to be transferred to metropolitan hospitals. Some regional hospitals have no on-site TB specialist medical practitioners, and

because of the distance from the hospital, there are very few opportunities for home visits from Victorian Tuberculosis Program specialist nursing staff, so regional patients must rely on telephone or video consultations for their diagnostic and follow-up consultations as well as their treatment supervision visits. The Victorian Tuberculosis Program (VTP) is Victoria's state-wide provider and coordinator of tuberculosis control.

In regional Victoria, the number of people born overseas is increasing. For example, in the 2006 census, there were 1,964 Indian-born people recorded as living in regional Victoria, which grew to 8,592 persons in 2016; similarly, the Philippines-born population was 2,700 in 2006 and 6,085 in 2016 [2]. The Australian Government has made changes to Commonwealth immigration policy intended to stimulate economic growth outside metropolitan areas in recent years. These various changes are focused on attracting migrants and international students to regional areas [3]. Historically, most migrants to Victoria have settled in the capital city of Melbourne, with many coming from countries with high TB incidence, such as India, the Philippines, and Sudan [4,5]. Such changes to policy influence migration patterns and may impact the distribution of TB cases within Victoria, which may also have implications for optimising health service delivery models [5].

TB incidence in Victoria remains low, with 436 TB cases notified in 2018, representing 6.9 cases per 100,000 population [6]. In Australia, research to date has tended to focus on metropolitan areas, where case numbers typically predominate. Understanding TB treatment delays among regional patients provides important insights into VTP performance and is a critical step towards tuberculosis elimination. Globally, TB surveillance data have been recognised as an important data source for assessing the disease burden and epidemiological trends in TB [7]. Evaluating treatment outcomes and delays in regional

areas will inform practice and policy. We aimed to describe notified TB cases in regional Victoria from 1995 to 2019, including trends and outcomes over these 24 years.

Methods

Data Source

We used routinely collected TB surveillance data. Data for all notified active tuberculosis cases in Victoria are collected by the VTP nurse consultants. Data are stored electronically in the Public Health Events Surveillance System (PHESS). PHESS is a centralised surveillance database containing data on all notifiable diseases in Victoria since 1991 [6]. The notification of active tuberculosis cases is mandatory in Victoria under the Public Health and Wellbeing legislation [6]. PHESS has standardised data collection templates to ensure consistency. Nurse consultants record patient demographic, clinical data and TB contacts in PHESS.

Data on the estimated resident population for all local government areas were obtained from the Australian Bureau of Statistics (ABS). The estimated resident population is the official figure of Australia's population based on the concept of "usual residence" and refers to all people, regardless of nationality or citizenship, who usually live in Australia, except foreign diplomatic personnel and their families [8].

Study design

We conducted a respective cohort study. Patients notified to the Australian department of health with active TB from 1995 to 2019 were identified. We analysed the data of these patients from the time they first developed TB symptoms until they completed TB treatment. Our study adhered to the Strengthening The Reporting of Observational Studies in Epidemiology (STROBE) guidelines (see S7 Table).

Study setting

The fieldwork for this study was conducted in Victoria by the VTP staff. Funded by the Victorian Government Department of Health, VTP is a centralised program located in metropolitan Victoria and works in partnership with hospitals and clinics in managing tuberculosis. All tuberculosis patients in Victoria are supervised by VTP nurse consultants [9,10].

Study Population

The population for this study included all people of all ages who had been diagnosed with tuberculosis and notified to the department of health.

Inclusion criteria.

Patients were included in the study if they met the following inclusion criteria:

1. Diagnosed with TB in Victoria and notified to the Victorian department of health from 1 January 1995 to 31 December 2019. TB cases were defined in accordance with a standard national case definition based on either laboratory definitive evidence requiring isolation of Mycobacterium tuberculosis complex by culture or nucleic acid testing or clinical diagnosis accompanied by treatment [11].
2. Having received tuberculosis treatment in Victoria.

Exclusion criteria.

Patients were excluded from the study if they:

1. were notified before 1995 or after 2019
2. Lacking residential addresses

Variables

The dependent variables were the treatment delays and the treatment outcomes (study outcomes). Treatment outcomes included completed treatment, lost to follow up, died of TB, died of other causes during treatment for TB, and transferred interstate or overseas. For the treatment delays, we adapted the definitions outlined by Van Wyk et al. [12], which proposed that: 'Patient treatment delay' is the period (in the number of days) between the onset of any self-reported TB symptoms and the first visit to a health care facility. 'Health system delay' is the period (in the number of days) between the first health care facility visit and initiation of TB treatment. 'Diagnostic delay' is defined as the period (in the number of days) between the onset of any self-reported TB-related symptoms and the time a chest x-ray was performed. 'Treatment initiation delay' was the period between a positive specimen (TB confirmed) and treatment initiation.

We also extracted the following independent variables from PHESS, (1) demographic data: age (age groups in years), sex (male or female), country of birth (name of the country), Aboriginal and Torres Strait Islander status (Aboriginal and/or Torres Strait Islander or not Aboriginal and/or Torres Strait Islander), local government areas (local government area), self-reported residency status (Australian-born, permanent resident, refugee/humanitarian, visitor, overseas student, other and unknown status), and for overseas-born cases, year of arrival in Australia (year). (2) Clinical characteristics: year of tuberculosis notification (year), the manifestation of tuberculosis (pulmonary, extrapulmonary or both), chest X-ray results (abnormal, cavitation or normal), laboratory results (smear, culture, or gene expert), and treatment outcome (died of TB, died of other causes during treatment for TB, completed treatment, lost to follow up, and transferred interstate or overseas).

Ethical considerations

Approval from a Human Research Ethics Committee for this study was not required as the data were collected for the purposes of public health action, as defined in the Public Health and Wellbeing Act 2008 and were considered as being for quality assurance and auditing purposes. Patients were informed of the purpose of data collection and consented to their data being used for tuberculosis surveillance and medical research at the time of collection. All data were fully anonymised during the data extraction process. For example, participants' names, phone numbers, addresses, and birth dates were removed. Patient identification numbers, postcodes, and gender were coded. Age was changed to age group. In the publications that come from this study, patients will remain anonymous.

Data analysis

Data cleaning and analyses were conducted using STATA version 14.

Managing missing data

We used a listwise deletion method when missing data contained residential addresses (participants were allocated neither to regional nor metropolitan areas) because our exposure of interest was regionality. When missing data did not have the key variable (i.e., residential address), we utilised the pairwise deletion approach, which allowed us to retain data and reduce the possibility of selection bias.

Analysis

Descriptive and multivariable analyses were performed. Incidence rates were calculated using the mid-year estimated resident population. Pearson's x^2 test was used to test the association between categorical variables. A two-tailed p-value of <0.05 was considered statistically significant. In logistic regression, we compared complete treatment with death, irrespective of the cause, lost to follow-up and transferred interstate or overseas. Died of TB was compared with completed treatment, lost to follow-up, died of other causes during treatment for TB, and transferred interstate or overseas.

We included all independent variables in all multivariable analyses because we believed they could all affect the outcomes. Our variable of interest was regionality. The proportional-hazards assumption was assessed using Kaplan-Meier survival curves by including time-dependent covariates in the model and with Schoenfeld residuals. In cases where proportionality assumptions were not met, analyses were stratified. Kaplan-Meier survival curves were used to show various delays in presentation, diagnosis, and treatment between regional and metropolitan cohorts, and Cox proportional hazard analyses were performed to assess these delays. Patient follow-up was censored, 1. at the first visit to a health care facility (Patient treatment delay), 2. at the initiation of TB treatment (Health system delay), 3. at the time a chest x-ray was performed (Diagnostic delay), 4. at the treatment initiation (Treatment initiation delay).

Because of limited previous data, treatment outcomes were analysed using data from 2005 to 2019, and for the analysis of treatment delays, we used data from 2012 to 2019.

Results

A total of 8,819 TB cases were notified to the Victorian Government Department of Health between 1995 and 2019. Among the 8,819 cases, 611 (7%) were recorded in regional areas, 8,163 (93%) in metropolitan areas of Victoria and 45 (1%) had neither regional nor metropolitan residential addresses (see Fig 1). Forty-five cases with no residential addresses were excluded from the study as they were classified as neither regional nor metropolitan. Of the 611 people in regional areas, 343 (56%) were male, 401 (66%) were overseas-born, and for 10 cases (2%), there was no country of birth recorded. Among the 8,163 TB cases in metropolitan areas, 4,316 (53%) were male, 8 (0.1%) had no gender reported and 7,375 (90%) were overseas-born.

Fig 1. The flow of patients through the study

Data recorded before 2005 had missing treatment outcomes for many cases and were therefore excluded from the analysis of treatment outcomes (Fig 1). The overall treatment completion rates were similar among the regional and metropolitan cohorts: 85% and 90%, respectively.

Table 1 describes the characteristics of notified TB cases in Victoria from 1995 to 2019 by location of residence and birth. The proportion of overseas-born cases in the regional cohort was 66% compared to 90%, in metropolitan areas. The 25 to 34 age group had the largest proportion of cases in both regional and metropolitan areas. In this age bracket, there were 27% of overseas-born and 8% of Australian-born cases in regional settings, and 31% of overseas-born and 11% of Australian born cases in metropolitan areas. The proportions of multidrug-resistant TB (MDR-TB) cases among the regional and metropolitan patients were

similar. The four MDR-TB cases reported in the regional cohort all occurred in people born overseas. In the metropolitan cohort, the proportion of MDR-TB cases was the same (1%) amongst overseas and Australian-born persons. Extensive drug-resistant TB (0.03%) and genotypic rifampicin-resistant TB (0.04%) were only reported in the metropolitan area among overseas-born cases.

Table 1. Characteristics of the notified cases of Tuberculosis in Victoria from 1995 to 2019.

Variable		Regional (n = 601)				Metropolitan (n = 8,163)			
		Overseas-born (n = 401)		Australian-born (n = 200)		Overseas-born (n = 7,375)		Australian born (n = 788)	
		Total	Proportion %	Total	Proportion %	Total	Proportion %	Total	Proportion %
Gender	Male	215	54	123	62	3,869	52	447	57
	Female	186	46	77	39	3,500	47	339	43
	Unknown	0	0	0	0	6	0	2	0
Age group	Under 5	3	1	11	6	31	0	115	15
	5-14	12	3	7	4	122	2	79	10
	15-24	49	12	14	7	1,303	18	129	16
	25-34	110	27	15	8	2,272	31	87	11
	35-44	60	15	10	5	1,188	16	60	8
	45-54	49	12	15	8	715	10	67	9
	55-64	33	8	31	16	562	8	56	7
	65 and above	85	21	97	49	1,181	16	195	25
	Unknown	0	0	0	0	1	0	0	0
Manifestation	Pulmonary	193	48	131	66	2,864	39	459	58
	Pulmonary Plus other sites	42	10	18	9	936	13	117	15
	Extra Pulmonary	162	40	49	25	3,396	46	206	26
	Unknown	4	1	2	1	179	2	6	1
Susceptibility	Fully sensitive	233	58	117	59	4,376	59	402	51
	Multidrug - resistant tuberculosis	4	1	0	0	88	1	9	1
	Other resistance	12	3	2	1	379	5	29	4
	Extensively drug-resistant tuberculosis	0	0	0	0	2	0	0	0
	Genotypic Rifampicin resistant tuberculosis	0	0	0	0	3	0	0	0
	Unknown	152	38	81	41	2,527	34	348	44

Treat outcome (from 2005 to 2019	Completed treatment	238	86	88	82	4,491	90	442	92
	Lost to follow-up	5	2	4	4	106	2	8	2
	Died from other cause	12	4	12	11	128	3	22	5
	Died of tuberculosis	4	1	3	3	58	1	5	1
	Still on treatment at the time of data extraction	1	0	0	0	1	0	0	0
	Transferred interstate or overseas	18	6	0	0	222	4	1	0
	Unknown	0	0	0	0	1	0	0	0

Note: Treatment outcome regional, n = 385 and Metro, n = 5,485.

The number of tuberculosis cases in regional Victoria has fluctuated over time, with 129 notified from 1995 to 1999, 97 from 2000 to 2004, and 155 from 2015 to 2019. Table 2 compares the TB incidence rate between regional and metropolitan areas. From 1995 to 1999, there were 129 cases with a mean incidence rate of 2.0, 95% CI 1.3-2.7 per 100,000 population in regional Victoria, while in the metropolitan there were 1,271 cases with a mean incidence rate of 7.7, 95% CI 6.9-8.4. The TB incidence in regional and metropolitan areas is fluctuating; in the 2015 to 2019 period, the mean incidence for the regional cohort was 2.1, 95% CI 1.5-2.7 and 7.9, 95% CI 7.3-8.4 in the metropolitan.

Table 2. Tuberculosis incidence rate per 100,000 population in regional Victoria and metropolitan Victoria from 1995 to 2019.

Years	Regional areas		Metropolitan areas	
	Tuberculosis cases	Mean incidence rate per 100,000 population (95% CI)	Tuberculosis cases	Mean incidence rate per 100,000 population (95% CI)
1995-1999	129	2.0 (1.3-2.7)	1,271	7.7 (6.9-8.4)
2000-2004	97	1.5 (1.0-1.9)	1,407	8.0 (7.6-8.4)
2005-2009	98	1.4 (1.2-1.6)	1,742	9.1 (8.9-9.4)
2010-2014	132	1.8 (1.4-2.1)	1,841	8.7 (7.9-9.5)
2015-2019	155	2.1 (1.5-2.7)	1,902	7.9 (7.3-8.4)

TB cases among overseas-born people aged 20 to 49 years have rapidly increased since 2004 (Fig 2). Conversely, in the Australian-born population aged 20 to 49 years, cases have remained stable since 1995. The number of TB cases among Australian-born people aged ≥50 years has decreased from 1995 and slightly upturned since 2014.

Fig 2. Tuberculosis cases in regional Victoria by year of notification, age, and country of birth from 1995 to 2019.

A total of 386 overseas-born cases had their year of arrival in Australia recorded. Half of the people (197; 51%) developed TB disease within five years of arrival in Australia. Out of these 197 cases, 58 (29%) were permanent residents, 43 (22%) were refugees, 19 (10%) were visitors, 18 (9%) were overseas students, and 59 (30%) had unknown residential status. Among 19 overseas students, 18 developed TB within five years and one between six

and 11 years of arrival. Of the 46 refugees recorded in the study, 43 were diagnosed with TB within five years of arrival. The risk of developing TB remained for many years after people arrived in Australia; 10 (3%) people were diagnosed with TB after 53 years of arrival (see, Fig 3).

Fig 3. Notified cases of tuberculosis in the overseas-born people in regional Victoria from 1995-2019 by the number of years since arrival in Australia.

In 2016, the TB incidence rate for the Australian-born population was 0.5 per 100,000 people and 12.0 per 100,000 people born overseas. Thirteen frequently reported countries of birth for the overseas-born cases from 1999 to 2019 are shown in Fig 4. People born in these 13 countries make up 62% of the overseas-born cases during the study period. India had the highest number of notified cases, 58 (14%), followed by the Philippines, 48 (12%). People born in India and the Philippines accounted for 26% of all the TB cases in regional areas.

Fig 4. Notified cases of tuberculosis in regional Victoria for overseas-born people from 1999 to 2019, by country of birth.

Among 5,870 cases with known treatment outcomes, 5,259 (90%) completed treatment, 124 (2%) were lost to follow-up, 174 (3%) died of another cause while on TB therapy, 70 (1%) died of TB, 2 (0.03%) were still on treatment at the time of data extraction, and 241 (4%) were transferred either interstate or overseas. Table 3 shows univariable and multivariable analyses of predictors of treatment completion and dying of TB. Living in a regional area was associated with lower odds of treatment completion on univariable analysis (OR = 0.6, 95% CI 0.5-0.8, P = 0.002). After adjusting for the effect of age, sex, drug

susceptibility and country of birth in the model, living in a regional area remained significantly associated with lower treatment completion (Adjusted OR [AOR] = 0.7, 95% CI 0.5-0.9, P = 0.019). On multivariable analysis, older age and male sex were also predictors of lower treatment completion.

Living in a regional area did not significantly increase the odds of dying from TB on univariable analysis (OR = 1.6, 95% CI 0.7-3.5, P = 0.244). In a model that included all the five variables in the multivariable analysis, regionality was not associated with dying of TB (AOR = 1.8, 95% CI 0.7-4.2, P = 0.198). Older age was significantly associated with dying of TB.

Table 3. Univariable and multivariable analysis of predictors of treatment completion and dying of tuberculosis from 2005 to 2019.

Variable	Treatment completion				Died of tuberculosis			
	Univariable analysis		Multivariable analysis		Univariable analysis		Multivariable analysis	
	OR (95%)	P valve	AOR (95%)	P valve	OR (95%)	P valve	AOR (95%)	P valve
Regional (reference group: metropolitan cases)	0.6 (0.5-0.8)	0.002	0.7 (0.5-0.9)	0.019	1.6 (0.7-3.5)	0.244	1.8 (0.7-4.2)	0.198
Age ≥ 65 years (reference group: age < 65 years)	0.2 (0.2-0.3)	<0.0001	0.2 (0.2-0.3)	<0.0001	12.6 (7.6-20.9)	<0.0001	9.9 (5.8-16.9)	<0.0001
Male sex (reference group: female sex)	0.6 (0.5-0.7)	<0.0001	0.7 (0.6-0.8)	<0.0001	1.8 (1.1-3.0)	0.020	1.5 (0.9-2.6)	0.131
Drug resistant tuberculosis (reference group: fully sensitive tuberculosis)	0.8 (0.6-1.1)	0.228	0.8 (0.6-1.1)	0.128	1.7 (0.8-3.5)	0.179	1.9 (0.9-4.1)	0.104
Overseas-born (reference group: Australian-born	0.9 (0.7-1.2)	0.415	0.7 (0.5-1.1)	0.094	0.9 (0.4-1.8)	0.682	1.8 (0.7-4.9)	0.238

Notes: AOR is an adjusted odds ratio.

The effect of regionality in multivariable Cox proportional hazard model analyses is shown in Table 4. The model included age, sex, place of birth, place of residence, and drug susceptibility. Data prior to 2012 were incomplete in relation to TB symptom onset, chest X-

ray findings and laboratory investigations and were therefore unable to be included in the analyses of delay in TB diagnosis and treatment. A total of 241 cases in regional and 2,997 in metropolitan areas were included.

Patient and health system delays were similar in regional and metropolitan areas for cases with pulmonary involvement. In regional areas, people with pulmonary involvement underwent chest x-ray (diagnostic delay) slightly sooner than those notified in metropolitan areas (median six days versus nine days, AHR = 1.2, 95% CI 1.0-1.5, P = 0.047). Conversely, healthcare system delay was non-significantly longer in regional than in metropolitan patients (median 64 days versus 54 days, AHR = 0.8, 95% CI 0.6-1.0, P = 0.094).

Table 4. Adjusted relationship between regionality in Cox regression models of delays in the cascade of care among tuberculosis patients in Victoria from 2012 to 2019.

			Effect of regional in Cox regression analysis			
Time period outcome	Regional area Median (interquartile range) days	Metropolitan area Median (interquartile range) days	Number observed in regional	Number observed in metropolitan	Adjusted Hazard ratio (95% CI)	P-value
Patient with pulmonary involvement						
Patient delay	21 (1-76)	24 (1-67)	113	1,293	0.9 (0.8-1.1)	0.389
Health system delay	21.5(7-44)	25 (7-65)	146	1,691	1.2 (1.0-1.4)	0.102
Diagnostic delay: Presentation to First chest-x-ray	6 (0-27)	9 (0-41)	122	1,343	1.2 (1.0-1.5)	0.047

Treatment delay: First chest X-ray to start of tuberculosis treatment	8 (3-23)	9 (3-29)	131	1,565	1.0 (0.9-1.3)	0.614
Extrapulmonary patients						
Patient delay	11.5 (0-68)	23 (0-75)	62	907	1.0 (0.7-1.2)	0.756
Health system delay	64 (26-137)	54 (21-112)	78	1,107	0.8 (0.6-1.0)	0.094
Diagnostic delay: Presentation to First chest x-ray	26 (5-92)	30 (5-72)	47	798	0.9 (0.7-1.2)	0.393
All patients						
Treatment delay 2: specimen test to treatment initiation	7 (1-19)	5 (1-17)	151	1,996	1.0 (0.8-1.2)	0.747

The Kaplan-Meier curves for patient delay are shown in Fig 5.

Fig 5. Kaplan-Meier curves in tuberculosis patients in Victoria, Australia from 2012 to 2019.

Discussion

We report the trends and treatment outcomes of notified TB cases in regional areas of Victoria, Australia, from 1995 to 2019. The incidence of TB is low in regional areas, and this is consistent with the findings from studies reported in the United States of America (USA)

and the United Kingdom. For example, a study in Appalachia, USA, reported that in 2005 the rate of tuberculosis in regional Appalachia was 2.1 compared to 2.7 per 100,000 population in metropolitan areas [13]. A similar study conducted in England and Wales from 2001 to 2003 found that the rate of TB in metropolitan areas was 6.3-fold higher compared to regional areas [14].

We observed that there had been a slight increase in TB incidence in regional Victoria from 1995 to 2019. The increase in TB incidence in regional Victoria is not surprising considering the increase in people born in high TB incidence countries [2]. In the regional cohort, the proportion of TB among the overseas-born population was twice that of the Australia-born people. There is an increase in TB cases aged between 20 and 49 years among the overseas-born in the regional cohort. These results suggest there may be a public health benefit in increasing latent TB detection and treatment in regional areas, targeting 20-49-year-old overseas-born people, and offering TB preventative therapy to those found with latent TB.

In regional Victoria, TB resistance was more common in overseas-born cases, consistent with other Australian studies [6,15]. We analysed the time from arrival in Australia to TB diagnosis for overseas-born cases in the regional cohort. More than half of the overseas-born cases were notified within five years of arrival in Australia. The high TB notification within the first five years of arrival may be attributed to the latent TB reactivation [16]. It is worth noting that the majority of the refugees were diagnosed within five years of arrival. This may be a result of more intensive screening soon after arrival, including testing for LTBI in asylum seekers but not migrants more generally. Refugees may also return overseas less frequently than other migrants and be less likely to be reinfected.

In this study, health-seeking behaviour and treatment outcomes were similar between the regional and metropolitan settings. These results suggest that existing programs are functioning well, although the possible trend toward health service delays requires further monitoring and reviewing opportunities for programmatic strengthening. In addition, people aged over 64 years are at significantly greater risk of dying from TB and, in appreciation of this risk, more intensive care may be required.

Due to a paucity of research in regional areas of countries with a low incidence of TB, we cannot make a direct comparison between our study and other published literature. Putting aside the comparison between regional and metropolitan data, our results in relation to delays in TB diagnosis and treatment are consistent with other Australian studies as well as systematic reviews [10,17,18]. For example, Bello et al., [17] performed a systematic review of 198 studies. They reported a median duration of patient delay of 28 days and a health system delay of 18 days compared to 21 days for each of these categories for the regional patients in our study. Of interest, extrapulmonary tuberculosis in our cohort had a much longer health system delay, averaging 64 days for regional patients.

Limitations

Strengths of this study include the use of a comprehensive central database that includes important demographic, clinical and laboratory data, allowing for the incorporation of other factors outlined in this manuscript and a long study period of 25 years. However, we acknowledge that data for the entire study period are not available for all data fields (e.g., treatment outcomes, health system delays), limiting trend analysis. Some of the data in our study, such as dates of symptom onset and healthcare presentation, were collected retrospectively from patients and thus may contain inaccuracies relating to recall bias. Data

on some factors that could have influenced the treatment delay and outcomes, such as educational level was limited.

Conclusion

Tuberculosis in regional Victoria is more common among the overseas-born population, and patients with extrapulmonary TB in regional areas have non-significant minor delays in treatment commencement. Increasing migration from high incidence TB countries to regional settings in Australia requires an ongoing review of available and accessible health services to limit delays in timely diagnosis and treatment. Increasing access to LTBI management and enhanced diagnostic pathways in regional areas may assist in reducing the burden and impact of TB in the future.

Author Contributions

Nompilo Moyo: Conceptualization, Formal analysis, Methodology, Project administration, Writing – original draft.

Tay EL: Data curation, Writing – review & editing.

Trauer JM, Burke L, Boyd SC, Singh KP, Jackson J, Commons RJ: Writing – review & editing.

Denholm JT: Conceptualization, Supervision, Writing – review & editing.

Acknowledgements

The authors would like to thank the clinical nurse consultants for collecting and entering data into the database.

References

1. Australian Bureau of Statistics. National, state and territory population. [Internet]. Australian Bureau of Statistics.; 2020 [cited 2020 Nov 30]. Available from: https://www.abs.gov.au/statistics/people/population/national-state-and-territory-population/latest-release

2. Australian Bureau of Statistics. 2016 Census Community Profiles. [Internet]. Australian Bureau of Statistics.; 2017 [cited 2020 Nov 30]. Available from: https://quickstats.censusdata.abs.gov.au/census_services/getproduct/census/2016/communityprofile/2RVIC?opendocument

3. Department of Home Affairs, Australia. Regional migration. [Internet]. Department of Home Affairs, Australia.; 2020 [cited 2020 Nov 30]. Available from: https://immi.homeaffairs.gov.au/visas/working-in-australia/regional-migration

4. Trauer JM, Williams B, Laemmle-Ruff I, Horyniak D, Caplice LVS, McBryde ES, et al. Tuberculosis in migrants to Australia: Outcomes of a national screening program. Lancet Reg Health West Pac. 2021;10:100135–100135.

5. McBryde ES, Denholm JT. Risk of active tuberculosis in immigrants: effects of age, region of origin and time since arrival in a low-exposure setting. Med J Aust. 2012;197(8):458–61.

6. Bright, A., Denholm, J., Coulter, C., Waring, J., & Stapledon, R. Tuberculosis notifications in Australia, 2015-2018. Commun Dis Intell. 2020;44:1–39.

7. World Health Organisation. Strengthening TB surveillance. [Internet]. 2022 [cited 2022 Jun 26]. Available from: https://www.who.int/westernpacific/activities/strengthening-tb-surveillance

8. Australian Bureau of Statistics. 3107.0.55.006 - Information Paper: Population Concepts, 2008 [Internet]. Australian Bureau of Statistics.; 2008 [cited 2021 Jan 12]. Available from: https://www.abs.gov.au/AUSSTATS/abs@.nsf/Lookup/3107.0.55.006Glossary12008?OpenDocument

9. Moyo N, Tay EL, Nolan A, Graham HR, Graham SM, Denholm JT. TB contact tracing for young children: an Australian cascade of care review. Public Health Action. 2021;11(2):91–6.

10. Dale KD, Tay EL, Trauer JM, Trevan PG, Denholm JT. Comparing tuberculosis management under public and private healthcare providers: Victoria, Australia, 2002-2015. BMC Infect Dis. 2017;17(1):324–324.

11. Australian Government Department of Health. Tuberculosis case definition [Internet]. Australian Government Department of Health; 2010 [cited 2022 Apr 12]. Available from: https://www1.health.gov.au/internet/main/publishing.nsf/Content/cda-surveil-nndss-casedefs-cd_tb.htm

12. VAN WYK SS, ENARSON DA, BEYERS N, LOMBARD C, HESSELING AC. Consulting private health care providers aggravates treatment delay in urban South African tuberculosis patients. Int J Tuberc Lung Dis. 2011;15(8):1069–76.

13. Wallace RM, Armstrong LR, Pratt RH, Kammerer JS, Iademarco MF. Trends in Tuberculosis Reported From the Appalachian Region: United States, 1993-2005. J Rural Health. 2008;24(3):236–43.

14. ABUBAKAR I, CROFTS JP, GELB D, STORY A, ANDREWS N, WATSON JM. Investigating urban–rural disparities in tuberculosis treatment outcome in England and Wales. Epidemiol Infect. 2007;136(1):122–7.

15. Toms C, Stapledon R, Waring J, Douglas P. Tuberculosis notifications in Australia, 2012 and 2013. Commun Dis Intell. 2015;39(2):E217–35.

16. Dale KD, Trauer JM, Dodd PJ, Houben RMGJ, Denholm JT. Estimating Long-term Tuberculosis Reactivation Rates in Australian Migrants. Clin Infect Dis. 2020;70(10):2111–8.

17. Bello S, Afolabi RF, Ajayi DT, Sharma T, Owoeye DO, Oduyoye O, et al. Empirical evidence of delays in diagnosis and treatment of pulmonary tuberculosis: systematic review and meta-regression analysis. BMC Public Health. 2019;19(1):820–820.

18. Williams E, Cheng AC, Lane GP, Guy SD. Delays in presentation and diagnosis of pulmonary tuberculosis: a retrospective study of a tertiary health service in Western Melbourne, 2011–2014. Intern Med J. 2018;48(2):184–93.

Tuberculosis notifications in regional Victoria, Australia: implications for public health care in a low incidence setting

Response to reviewer 2

Your feedback 1. The Methods section still requires revisions. Currently there are sub-sections which are not properly described. For example, the study design sub-section is just one short sentence. I suggest revising this section to include the following sub-sections in this particular order: Data Source, Study Population, Variables, and Statistical Analysis. Under the Data Source sub-section - describe the data source, study design and study setting. Under Study Population sub-section - describe the target population by age, inclusion and exclusion criteria and missing data. Under the Variables sub-section - describe the variables, dependent and independent variables. Under Statistical Analysis sub-section - describe the analyses conducted in the study.

Our response. We have organised the methods section under the suggested sub-sections. See revised lines 99 to 167.

Response to reviewer 3

Your feedback 1: Lines 30-32: the methods section of the abstract is quite brief missing important details that will help readers to understand the results presented from a survival analysis. Sample size, follow-up, date variables, outcomes, censoring, and statistical analysis issues are missing.

Our response. We have amended the abstract, and it now reads, "Background: Regionality is often a significant factor in tuberculosis (TB) management and outcomes worldwide. A wide range of context-specific factors may influence these differences and change over time. We compared TB treatment in regional and metropolitan areas, considering demographic and temporal trends affecting TB diagnosis and outcomes. Methods: Retrospective analyses of data for patients notified with TB in Victoria, Australia, were conducted. The outcomes were treatment delays and treatment outcomes. Multivariable

Cox proportional hazard model analyses were performed to investigate the effect of regionality in the management of TB. Six hundred and eleven (7%) TB patients were notified in regional and 8,163 (93%) in metropolitan areas between 1995 and 2019. Of the 611 cases in the regional cohort, 401 (66%) were overseas-born. Fifty-one percent of the overseas-born patients in regional Victoria developed TB disease within five years of arrival in Australia. Four cases of multidrug-resistant tuberculosis were reported in regional areas, compared to 97 cases in metropolitan areas. A total of 3,238 patients notified from 2012 to 2019 were included in the survival analysis. Patient follow-up was censored at the first visit to the health care facility (Patient treatment delay) and at the initiation of TB treatment (Health system delay). Patient, health system, and treatment delays were similar in regional and metropolitan areas for cases with pulmonary involvement. Cases with extrapulmonary TB in regional areas have a non-significantly longer healthcare system delay than patients in metropolitan (median 64 days versus 54 days, AHR = 0.8, 95% CI 0.6-1.0, P = 0.094).

Conclusion: Tuberculosis in regional Victoria is common among the overseas-born population, and patients with extrapulmonary TB in regional areas experienced a non-significant minor delay in treatment commencement with no apparent detriment to treatment outcomes. Improving access to LTBI management in regional areas may reduce the burden of TB." Lines 26-49.

Your feedback 2. Line 36-39-->the statement in these lines feels like labeling. In the absence of adequate number of cases, it is difficult to associate multidrug resistant TB and being overseas born. Furthermore, a statement in line 36 reads, 'the proportion of MDR-TB cases in regional vs metropolitan areas is similar'. In the next line, however, it presents only four cases of MDR-TB in regional vs 97 in metropolitan. The statements in the lines indicated above are difficult to follow◊present them consistently in terms of proportion or in absolute

numbers. The data presented in Table 1 of the body of the document, do not support this statement. 4 MDR-TB vs 0 in regional and metropolitan--> this data is not adequately powered to support the statement provided in these lines.

Our response. We have amended the statement and it now reads "Four cases of multidrug-resistant tuberculosis were reported in regional areas, compared to 97 cases in metropolitan areas." Lines 37-38.

Your feedback 3. Lines 40-44: "Cases with extra pulmonary TB in regional areas have a non-significantly longer healthcare system delay than patients in metropolitan (median 64 days versus 54 days, AHR = 0.8, 95% CI 0.6-1.0, P = 0.094). People living in regional areas have a non-significantly higher odds of dying of TB (AOR = 1.8, 95% CI 0.7-4.2, P = 0.198)."
In the above text, the authors presented mixed effect sizes, AHR vs AOR. However, the effect estimate from the Cox proportional hazards region is expressed in HRs than ORs. The other thing is that the authors should clearly specify their outcome of interest than generally providing 'TB mgt. outcome'.

Our response. We have amended the abstract, please refer to our response 1. Lines 26-49.

Your feedback 4. The other thing is that the authors should clearly specify their outcome of interest than generally providing 'TB mgt. outcome'.

Our response. We have added the following statement to the abstract and methods sections: "The study outcomes were treatment delays and treatment outcomes". Lines 31, 144-145.

Your feedback 5. While information presented in the background is critical to understand context of the problem, its nature, efforts to reduce the extent of the problem, challenges, gaps, and the need to conduct the current study, it is only presented in 18 lines missing important details. Therefore, i suggest the authors to consider adding a few details to give insight to the problem studied.

Our response. Thank you for this suggestion. We have added the following statement: “Understanding TB treatment delays among regional patients provides important insights into Victorian TB programme performance and is a critical step towards tuberculosis elimination. Globally, TB surveillance data have been recognised as an important data source for assessing the disease burden and epidemiological trends in TB (World Health Organization, 2022). Evaluating treatment outcomes and delays in regional areas will inform practice and policy.” Lines 90-95.

Your feedback 6. Data analysis- Line 139: consider here too the comments provided in the abstract regarding data analysis. Lines 150-153: present the global test results and also for the independent variables to attest that the proportional hazard regression was met.

Our response. We have amended the data analysis, and it now reads: “Descriptive and multivariable analyses were performed. Incidence rates were calculated using the mid-year estimated resident population. Pearson’s x^2 test was used to test the association between categorical variables. A two-tailed p-value of <0.05 was considered statistically significant. In logistic regression, we compared complete treatment with death, irrespective of the cause, lost to follow-up and transferred interstate or overseas. Died of TB was compared with completed treatment, lost to follow-up, died of other causes during treatment for TB, and transferred interstate or overseas. We included all independent variables in all multivariable analyses because we believed they could all affect the outcomes. However, our variable of interest was regionality. The proportional-hazards assumption was assessed using Kaplan-Meier survival curves by including time-dependent covariates in the model and with Schoenfeld residuals. In cases where proportionality assumptions were not met, analyses were stratified. Kaplan-Meier survival curves were used to show various delays in presentation, diagnosis, and treatment between regional and metropolitan cohorts, and Cox proportional hazard analyses were performed to assess these delays. Patient follow-up was censored, 1. at the first visit to a health care facility (Patient treatment delay), 2. at the initiation of TB treatment (Health system delay), 3. at the time a chest x-ray was performed (Diagnostic delay), 4. at the treatment initiation (Treatment initiation delay). Because of limited previous data, analyses of treatment outcomes were conducted using data from

2005 to 2019, while analyses of treatment delays used data from 2012 to 2019." Lines 190-210.

Your feedback 7. Results- Line 158: the 7% and 93% reported cases of TB do not reflect that 45 cases did not have residential information regarding their affiliation to regional of metropolitan.

Our response: We have amended this statement, and now it reads; "A total of 8,819 TB cases were notified to the Victorian Government Department of Health between 1995 and 2019. Among the 8,819 cases, 611 (7%) were recorded in regional areas, 8,163 (93%) in metropolitan areas of Victoria and 45 (1%) had neither regional nor metropolitan residential addresses (see Fig 1). Forty-five cases with no residential addresses were excluded from the study as they were classified as neither regional nor metropolitan." Lines 215-219.

Your feedback 8. Line 164-65: it is good to present the number of cases excluded. Or preferably provide the progress of pts. in a flow diagram.

Our response. Thank you for this suggestion. We have now provided the flow of patients through the study, see Fig 1. Line 224.

Your feedback 9. Line 175-177: this result has not been well reflected in the abstract.

Our response. Lines 175-177 were referring specifically to issues related to missing data. However, the abstract has been amended to more clearly reflect the overall findings as outlined above. Lines 26-49.

Your feedback 10. Table 1: the font size of contents of the table is significantly different from the text in the body.

Our response. We have increased the font size on all tables to 11 points.

Your feedback 11. The authors also consider avoiding in places where the total add to hundred or cell values added to the sample in respective subgroup. Or consider using '0' or NA to represent 'not available'

Our response. The tables have been amended accordingly.

Your feedback 12. Discussion- Owning to the arrival of overseas born individuals from high TB burden countries to Australia, there could be an active search for TB among this particular group which may introduce a diagnostic suspicion bias. Was there an effort in this study to exclude that diagnostic suspicion bias was not an issue or was there an effort to reduce it if there was any?

Our response. Thanks for raising this point, which we agree may be important in many contexts. We do not believe that this is a significant issue in our study, as the considerable majority of migration-associated testing for TB an active case finding occurs prior to visa issuing in countries of origin and are thus not reflected in these TB cases presented here. Overall, then, we do not account further for diagnostic suspicion bias during the study period.

Reference

World Health Organisation. (2022). Strengthening TB surveillance. World Health Organisation. https://www.who.int/westernpacific/activities/strengthening-tb-surveillance
