# Supplementary material for: Tuberculosis notifications in regional Victoria, Australia: Implications for public health care in a low incidence setting
Source: PLoS One. 2023 Mar 21;18(3):e0282884. doi: 10.1371/journal.pone.0282884 (PMC10030020; doi:10.1371/journal.pone.0282884)
Supplement: Attachment — Submitted filename: Response to Reviewers.docx [file pone.0282884.s002.docx]

Tuberculosis notifications in regional Victoria, Australia: implications for public health care in a low incidence setting

**Response to the reviewers’ comments**

To the Editor,

Thank you for the opportunity to revise this manuscript in light of the reviewer’s comments. The detailed responses to the comments are below, and the revised manuscript is attached. Our responses to the reviewer’s comments and the changes we made to the manuscript are written in red.

Our data will be accessible via <https://www.datadryad.org/stash/dashboard>. We will provide you with the DOI once the data has been deposited.

Please let us know if any further information would be helpful for you in evaluating this manuscript.

Regards,

Nompilo Moyo

**Reviewer 1**

# 1. Your feedback. The analyses are relatively straightforward but cover an important topic related to an important infectious disease (TB). The findings are relevant to understanding dynamics of tuberculosis in Victoria, Australia as well as potentially in other parts of Australia and other countries with similar population dynamics and underlying epidemiological conditions. However, as currently submitted the presentation of some of the findings as well as the unacceptable preponderance of typographical and grammatical errors need to be addressed. Some more specific comments are included below:

# Our response: Thank you for these comments, which we have responded to in detail below.

# 2. Your feedback. Line 59 – Consider defining what “regional” means in the context up front. The implication is that it refers to non-metropolitan areas but that will not become completely lear to non-Australian audiences until lines 112-115.

**Our response.** We have moved the definition of regional into introductory material, and the statement now reads: “Victoria is divided into two distinct geographic areas: metropolitan and regional. Metropolitan is the geographical area that defines Melbourne as the city and capital of the state of Victoria and has 31 local government areas. Regional is defined as a geographical area outside Melbourne city and includes 48 (out of a total of 79) local government areas from across the state [2]. Based on 2016 census data, 4,485,211 of Victoria’s total population of 5,926,624 lived within Melbourne, with less than 25% of the population living regionally (3). This equates to an average population density of 500 people per square kilometre in metropolitan Victoria compared to an average of 6 people per square kilometre in regional Victoria.” Lines 59-67.

# 3. Your feedback. Line 66-67 – is there a reference to back up the statement that many migrants arrive from countries known to have a high TB incidence?

**Our response.** We have added the reference and the statement now reads “Historically, most migrants to Victoria have settled in the capital city of Melbourne, with many coming from countries with high TB incidence, such as India, the Philippines, and Sudan (5, 6).” Lines 85-87.

# 4. Your feedback. Line 72-74 – it seems as if more was done that to describe notified TB cases…

**Our response.** We have provided a description of notified TB cases by region in Table 1. It’s not clear to us from this comment that changes to our manuscript are requested, but if specific additional information would be helpful editorially, we would be happy to consider it.

**5. Your feedback.** Line 128 – Ethical ConsiderationS

**Our response.** We have corrected the text and it now reads “Ethical Considerations” (Line 156).

**6. Your feedback.** Line 131 – should be “were considered” since this refers to data.

**Our response.** "Was considered" has been replaced with "were considered." Line 159.

**7. Your feedback.** Line 135 – “and” missing.

**Our response.** The sentence was amended to “For example, names, phone numbers, addresses, and dates of birth of participants were removed.” Lines 162-163.

**8. Your feedback.** Line 159 – excessive “and”

**Our response.** We amended the statement, and it now reads “Forty-five cases were excluded from the study because they lacked residential addresses and were classified as neither regional nor metropolitan.” Lines 195-197.

**9. Your feedback.** Line 169-170 – the overseas cases proportions are already mentioned in Lines 161-3

**Our response.** We reported two different proportions in these statements. The proportions reported in lines 161-3 were the overall proportions of the two cohorts, whereas the proportions described in lines 169-170 were specific to drug susceptibility. Lines 210-216.

**10. Your feedback.** Table 1 – formatting with some proportions rounded to whole numbers and other to one or two decimal points.

**Our response.** We have rounded the numbers in table 1 to whole numbers.

**11. Line 189-190** – it misrepresents the trend to say that there was a slight increase in TB notification over time. It is true when comparing the two time points but over time it actually fluctuated quite a bit including notable decreases over a few of the time periods (see Table 2)

**Our response.** We amended the text, and it now reads “The number of tuberculosis cases in regional Victoria has fluctuated over time, with 129 notified from 1995 to 1999, 97 from 2000 to 2004, and 155 from 2015 to 2019.” Lines 238-239.

**12. Your feedback.** Line 208-209 – median time to diagnosis AFTER ARRIVAL. Line 209-210 – In this particular case it is repetitive of the median cited in the prior sentence to say that half developed TB disease within 5 years (this is the Median)

**Our response.** The statement “The median time to diagnosis was five years, and the interquartile range was 1 to 17 years” has been deleted.

**13. Line 234-235** – Specify the directionality of the association (in this case an association between living in a regional area and lower odds of treatment completion).

**Our response:** We have amended the statement and it now reads “Living in a regional area was associated with lower odds of treatment completion on univariable analysis (OR = 0.6, 95% CI 0.5-0.8, P = 0.002).” Lines 283-285.

**14. Your feedback.** Line 290 – reference for latent TB reactivation?

**Our response:** We have added a reference to support the statement. The high TB notification within the first five years of arrival may be attributed to latent TB reactivation (15). Lines 348-349.

**15. Your feedback.** Figure 2 - Needs to be reworked entirely. For example, it is inappropriate to have stacked bars adding up to 200%. In addition, the data labels can be presented in a less crowded, more easily legible way.

**Our response.** Figure 2 has been amended. The residency status and the percentages in the data labels have been deleted. Figure 2 now shows the notified cases of tuberculosis in overseas-born people in regional Victoria from 1995-2019 by the number of years since arrival in Australia.

**Fig 2. Notified cases of tuberculosis in overseas-born people in regional Victoria from 1995-2019 by the number of years since arrival in Australia.**

| Time since arrival in Australia (Years) | Notified cases |
| --- | --- |
| 0-5 | 197 |
| 6-11 | 68 |
| 12-17 | 25 |
| 18-23 | 13 |
| 24-29 | 8 |
| 30-35 | 19 |
| 36-41 | 12 |
| 42-47 | 14 |
| 48-53 | 20 |
| 54 ≥ | 10 |

**Reviewer 2**

Background
**1. Your feedback.** Line 59: Authors introduce the term “regional Victoria”.
Comment: I suggest authors add a description what this means as it will be helpful for readers who are not familiar with Australia.

**Our response:** We have amended the manuscript to reflect this feedback. The manuscript now reads “Victoria is divided into two distinct geographic areas: metropolitan and regional. Metropolitan is the geographical area that defines Melbourne as the city and capital of the state of Victoria and has 31 local government areas. Regional is defined as a geographical area outside Melbourne city and includes 48 (out of a total of 79) local government areas from across the state [2]." Lines 59-67.

**2. Your feedback.** Line 65-67: “Historically, most migrants to Victoria have been based in the capital city of Melbourne, with many arriving from countries known to have a high TB incidence”.
Comment: Authors should mention examples of these countries and add references. Philippines, India, and Vietnam.

**Our response.** We have added the countries and supported the statement with a reference. This now reads: “Historically, most migrants to Victoria have settled in the capital city of Melbourne, with many coming from countries with high TB incidence, such as India, the Philippines, and Sudan [5, 6].” Lines 85-87.

**3. Your feedback.** Line 67-69: “Such changes to policy influence migration patterns and may impact the distribution of TB cases within Victoria”.
Comment: Please add a reference to support the claim of migration and TB cases.

**Our response.** We have added the reference to support the statement. The manuscript now reads “Historically, most migrants to Victoria have settled in the capital city of Melbourne, with many coming from countries with high TB incidence, such as India, the Philippines, and Sudan [5,6].” Lines 85-87.

**Methods**
**1. Your feedback**. Authors should consider improving this section by adding clear sub-sections, for example data source, study population, variables, statistical analysis etc.

**Our response.** Thank you for this suggestion. We have added these subsections to the methods section of the manuscript, most significantly reflected in lines 96-190.

**2. Your feedback.** Line 82-91: The description covered in line 82-91 seems to better fit elsewhere, either in the introduction or discussion sections and not in the methods section.

**Our response.** The description in line 82-91 has been moved to the Background section, and is now found in lines 69-78.

**3. Your feedback.** Line 100-106: The description of variables need to be improved. Indicate clearly the dependent and independent variables. Also, if variables are continuous or categorical and describe the categories. For example, sex (male or female), age (are there categories or continuous, age range?).

**Our feedback.** We have amended the paragraph and it now reads “We extracted the following variables: (1) demographic data: age (age groups in years), sex (male or female), country of birth (name of the country), Aboriginal and/or Torres Strait Islander status (Aboriginal and/or Torres Strait Islander or not Aboriginal and/or Torres Strait Islander), local government areas (local government area), self-reported residency status (Australian-born, permanent resident, refugee/humanitarian, visitor, overseas student, other or unknown status), and for overseas-born cases, year of arrival in Australia (year). (2) Clinical characteristics: year of tuberculosis notification (year), the manifestation of tuberculosis (pulmonary, extrapulmonary or both), chest X-ray results (abnormal, cavitation or normal), laboratory results (smear, culture, or gene expert), and treatment outcome (died of TB , died of other causes during treatment for TB, completed treatment, lost to follow up, and transferred interstate or overseas). The independent variables include age, gender, country of birth, and place of residency. Treatment outcomes and treatment delays are the dependent variables.” This information is now presented in a new section, in lines 131-144.

**4. Your feedback:** Description of the study population is missing, including inclusion or exclusion of observations and dealing with missing information.

**Our response.** We added the following text to the methods section of the manuscript: “We extracted TB surveillance data. Patients were included in the study if they met the following inclusion criteria: (1) diagnosed with TB in Victoria and notified to the Victorian department of health from 1 January 1995 to 31 December 2019, with TB cases defined in accordance with a standard national case definition based on either laboratory definitive evidence requiring isolation of Mycobacterium tuberculosis complex by culture or nucleic acid testing or clinical diagnosis accompanied by treatment [10] and (2) having received tuberculosis treatment in Victoria. Patients who were notified before 1995 or after 2019, along with those with missing residential addresses, were excluded from the study.” This information is now presented in a new section, in lines 107-115.

*Managing missing data*

We used a listwise deletion method when missing data contained residential addresses (participants were allocated neither to regional nor metropolitan areas) because our exposure of interest was regionality. When missing data did not contain the key variable (i.e., residential address), we utilised the pairwise deletion approach, which allowed us to retain data and reduce the possibility of selection bias.” This information is now presented in a new section, in lines 172-175.

**Results**
**1. Your feedback:** Line 160: description of exclusion of observations should be in the Methods section. Forty-five cases had no residential addresses recorded and were allocated neither to regional nor metropolitan areas and were excluded from the analysis.

**Our response:** We have amended the manuscript to reflect this feedback. This information is now presented in a new section, in lines 195-197.

**2. Your feedback:** Line 164-167: again, description of exclusion of observations should be in the Methods section

**Our response:** Data recorded before 2005 had missing treatment outcomes for many cases and were excluded from the analysis of treatment outcomes but was included in other analyses. A statement on how missing data were managed has been added to the methods. This information is now presented in a new section, in lines 170-175.

**3. Your feedback:** Line 199-200: table 2, the numbers are not properly formatted. Please revise.

**Our response:** We have formatted the numbers.

**4. Your feedback:** Line 243: Seems multivariable and multivariate are used interchangeably. Please maintain uniformity, use multivariable.

**Our response:** We have amended the manuscript accordingly.

**Discussion**
**1. Your feedback:** Line 289: “The higher TB rate among the overseas-born people could be attributed to latent TB reactivation”
Comment: Authors need to add references to support this statement.

**Our response:** We have added the reference. The high TB notification within the first five years of arrival may be attributed to latent TB reactivation (15). Lines 348-349.

**2. Your feedback:** Authors should add a study limitation section

**Our response:** We have added the limitations in the discussion section of the manuscript.

“Limitations

We acknowledge that data for the entire study period are not available for all data fields (e.g., treatment outcomes, health system delays), limiting trend analysis. Some of the data in our study, such as dates of symptom onset and healthcare presentation, were collected retrospectively from patients and thus may contain inaccuracies relating to recall bias. Data on some factors that could have influenced the treatment delay and outcomes, such as educational level was limited. Despite these limitations, the strengths of this study include the use of a comprehensive central database that includes important demographic, clinical and laboratory data, allowing for the incorporation of other factors outlined in this manuscript and a long study period of 24 years.” Lines 370-379.
