# Supplementary material for: Tuberculosis notifications in regional Victoria, Australia: Implications for public health care in a low incidence setting
Source: PLoS One. 2023 Mar 21;18(3):e0282884. doi: 10.1371/journal.pone.0282884 (PMC10030020; doi:10.1371/journal.pone.0282884)
Supplement: Attachment — Submitted filename: Responses to Plos one reviewers _R2.docx [file pone.0282884.s003.docx]

# **Tuberculosis notifications in regional Victoria, Australia: implications for public health care in a low incidence setting**

**Response to reviewer 2**

**Your feedback 1.** The Methods section still requires revisions. Currently there are sub-sections which are not properly described. For example, the study design sub-section is just one short sentence. I suggest revising this section to include the following sub-sections in this particular order: Data Source, Study Population, Variables, and Statistical Analysis. Under the Data Source sub-section - describe the data source, study design and study setting. Under Study Population sub-section - describe the target population by age, inclusion and exclusion criteria and missing data. Under the Variables sub-section - describe the variables, dependent and independent variables. Under Statistical Analysis sub-section - describe the analyses conducted in the study.

**Our response.** We have organised the methods section under the suggested sub-sections. See revised lines 99 to 167.

**Response to reviewer 3**

**Your feedback 1**: Lines 30-32: the methods section of the abstract is quite brief missing important details that will help readers to understand the results presented from a survival analysis. Sample size, follow-up, date variables, outcomes, censoring, and statistical analysis issues are missing.

**Our response.** We have amended the abstract, and it now reads, “Background: Regionality is often a significant factor in tuberculosis (TB) management and outcomes worldwide. A wide range of context-specific factors may influence these differences and change over time. We compared TB treatment in regional and metropolitan areas, considering demographic and temporal trends affecting TB diagnosis and outcomes. Methods: Retrospective analyses of data for patients notified with TB in Victoria, Australia, were conducted. The outcomes were treatment delays and treatment outcomes. Multivariable Cox proportional hazard model analyses were performed to investigate the effect of regionality in the management of TB. Six hundred and eleven (7%) TB patients were notified in regional and 8,163 (93%) in metropolitan areas between 1995 and 2019. Of the 611 cases in the regional cohort, 401 (66%) were overseas-born. Fifty-one percent of the overseas-born patients in regional Victoria developed TB disease within five years of arrival in Australia. Four cases of multidrug-resistant tuberculosis were reported in regional areas, compared to 97 cases in metropolitan areas. A total of 3,238 patients notified from 2012 to 2019 were included in the survival analysis. Patient follow-up was censored at the first visit to the health care facility (Patient treatment delay) and at the initiation of TB treatment (Health system delay). Patient, health system, and treatment delays were similar in regional and metropolitan areas for cases with pulmonary involvement. Cases with extrapulmonary TB in regional areas have a non-significantly longer healthcare system delay than patients in metropolitan (median 64 days versus 54 days, AHR = 0.8, 95% CI 0.6-1.0, P = 0.094).

Conclusion: Tuberculosis in regional Victoria is common among the overseas-born population, and patients with extrapulmonary TB in regional areas experienced a non-significant minor delay in treatment commencement with no apparent detriment to treatment outcomes. Improving access to LTBI management in regional areas may reduce the burden of TB.” Lines 26-49.

**Your feedback 2**. Line 36-39-->the statement in these lines feels like labeling. In the absence of adequate number of cases, it is difficult to associate multidrug resistant TB and being overseas born. Furthermore, a statement in line 36 reads, 'the proportion of MDR-TB cases in regional vs metropolitan areas is similar'. In the next line, however, it presents only four cases of MDR-TB in regional vs 97 in metropolitan. The statements in the lines indicated above are difficult to follow◊present them consistently in terms of proportion or in absolute numbers. The data presented in Table 1 of the body of the document, do not support this statement. 4 MDR-TB vs 0 in regional and metropolitan--> this data is not adequately powered to support the statement provided in these lines.

**Our response.** We have amended the statement and it now reads “Four cases of multidrug-resistant tuberculosis were reported in regional areas, compared to 97 cases in metropolitan areas.” Lines 37-38.

**Your feedback 3.** Lines 40-44: "Cases with extra pulmonary TB in regional areas have a non-significantly longer healthcare system delay than patients in metropolitan (median 64 days versus 54 days, AHR = 0.8, 95% CI 0.6-1.0, P = 0.094). People living in regional areas have a non-significantly higher odds of dying of TB (AOR = 1.8, 95% CI 0.7-4.2, P = 0.198)."
In the above text, the authors presented mixed effect sizes, AHR vs AOR. However, the effect estimate from the Cox proportional hazards region is expressed in HRs than ORs. The other thing is that the authors should clearly specify their outcome of interest than generally providing 'TB mgt. outcome'.

**Our response.** We have amended the abstract, please refer to our response 1. Lines 26-49.

**Your feedback 4.** The other thing is that the authors should clearly specify their outcome of interest than generally providing 'TB mgt. outcome'.

**Our response.** We have added the following statement to the abstract and methods sections: “The study outcomes were treatment delays and treatment outcomes”. Lines 31, 144-145.

**Your feedback 5.** While information presented in the background is critical to understand context of the problem, its nature, efforts to reduce the extent of the problem, challenges, gaps, and the need to conduct the current study, it is only presented in 18 lines missing important details. Therefore, i suggest the authors to consider adding a few details to give insight to the problem studied.

**Our response.** Thank you for this suggestion. We have added the following statement: “Understanding TB treatment delays among regional patients provides important insights into Victorian TB programme performance and is a critical step towards tuberculosis elimination. Globally, TB surveillance data have been recognised as an important data source for assessing the disease burden and epidemiological trends in TB (World Health Organization, 2022). Evaluating treatment outcomes and delays in regional areas will inform practice and policy.” Lines 90-95.

**Your feedback 6.** Data analysis- Line 139: consider here too the comments provided in the abstract regarding data analysis. Lines 150-153: present the global test results and also for the independent variables to attest that the proportional hazard regression was met.

**Our response.** We have amended the data analysis, and it now reads: “Descriptive and multivariable analyses were performed. Incidence rates were calculated using the mid-year estimated resident population. Pearson’s x^2^ test was used to test the association between categorical variables. A two-tailed p-value of <0.05 was considered statistically significant. In logistic regression, we compared complete treatment with death, irrespective of the cause, lost to follow-up and transferred interstate or overseas. Died of TB was compared with completed treatment, lost to follow-up, died of other causes during treatment for TB, and transferred interstate or overseas. We included all independent variables in all multivariable analyses because we believed they could all affect the outcomes. However, our variable of interest was regionality. The proportional-hazards assumption was assessed using Kaplan-Meier survival curves by including time-dependent covariates in the model and with Schoenfeld residuals. In cases where proportionality assumptions were not met, analyses were stratified. Kaplan-Meier survival curves were used to show various delays in presentation, diagnosis, and treatment between regional and metropolitan cohorts, and Cox proportional hazard analyses were performed to assess these delays. Patient follow-up was censored, 1. at the first visit to a health care facility (Patient treatment delay), 2. at the initiation of TB treatment (Health system delay), 3. at the time a chest x-ray was performed (Diagnostic delay), 4. at the treatment initiation (Treatment initiation delay). Because of limited previous data, analyses of treatment outcomes were conducted using data from 2005 to 2019, while analyses of treatment delays used data from 2012 to 2019.” Lines 190-210.

**Your feedback 7.** Results- Line 158: the 7% and 93% reported cases of TB do not reflect that 45 cases did not have residential information regarding their affiliation to regional of metropolitan.

**Our response:** We have amended this statement, and now it reads; “A total of 8,819 TB cases were notified to the Victorian Government Department of Health between 1995 and 2019. Among the 8,819 cases, 611 (7%) were recorded in regional areas, 8,163 (93%) in metropolitan areas of Victoria and 45 (1%) had neither regional nor metropolitan residential addresses (see Fig 1). Forty-five cases with no residential addresses were excluded from the study as they were classified as neither regional nor metropolitan.” Lines 215-219.

**Your feedback 8.** Line 164-65: it is good to present the number of cases excluded. Or preferably provide the progress of pts. in a flow diagram.

**Our response.** Thank you for this suggestion. We have now provided the flow of patients through the study, see Fig 1. Line 224.

**Your feedback 9.** Line 175-177: this result has not been well reflected in the abstract.

**Our response.** Lines 175-177 were referring specifically to issues related to missing data. However, the abstract has been amended to more clearly reflect the overall findings as outlined above. Lines 26-49.

**Your feedback 10.** Table 1: the font size of contents of the table is significantly different from the text in the body.

**Our response.** We have increased the font size on all tables to 11 points.

**Your feedback 11.** The authors also consider avoiding in places where the total add to hundred or cell values added to the sample in respective subgroup. Or consider using '0' or NA to represent 'not available'

**Our response.** The tables have been amended accordingly.

**Your feedback 12.** Discussion- Owning to the arrival of overseas born individuals from high TB burden countries to Australia, there could be an active search for TB among this particular group which may introduce a diagnostic suspicion bias. Was there an effort in this study to exclude that diagnostic suspicion bias was not an issue or was there an effort to reduce it if there was any?

**Our response.** Thanks for raising this point, which we agree may be important in many contexts. We do not believe that this is a significant issue in our study, as the considerable majority of migration-associated testing for TB an active case finding occurs prior to visa issuing in countries of origin and are thus not reflected in these TB cases presented here. Overall, then, we do not account further for diagnostic suspicion bias during the study period.

**Reference**

World Health Organisation. (2022). Strengthening TB surveillance. World Health Organisation. <https://www.who.int/westernpacific/activities/strengthening-tb-surveillance>
