# Supplementary material for: Tuberculosis notifications in regional Victoria, Australia: Implications for public health care in a low incidence setting
Source: PLoS One. 2023 Mar 21;18(3):e0282884. doi: 10.1371/journal.pone.0282884 (PMC10030020; doi:10.1371/journal.pone.0282884)
Supplement: Attachment — Submitted filename: Response to Plos one reviewers_V3. 2022.docx [file pone.0282884.s005.docx]

PONE-D-22-00893R2

Tuberculosis notifications in regional Victoria, Australia: implications for public health care in a low incidence setting

Reviewer #3:

Your feedback 1. I appreciate that the authors have considered the comments and improved the manuscript from the previous submission. However, I still request the authors to improve the background and discussion. In the background, the authors provided information that otherwise provided in the methods section under the subheading of study setting.

Our response. We have further amended the background and discussion sections of this manuscript to expand on context for the reader. The background section now reads: “Early diagnosis and treatment of tuberculosis are crucial to the effectiveness of TB control programmes (Yimer, et al., 2014). Previous studies have reported delayed TB diagnosis in countries with low incidence (Dale et al., 2017; Kelly et al., 2017; Labuda, 2022). For example, Labuda et al. (2022) conducted a study involving 21 patients and reported that eight (38%) had their TB diagnosis delayed by months. In a comparable study involving 34 patients with TB, Kelly et al. (2017) reported that 17 (50%) were diagnosed with other diseases, and the average time between admission and tuberculosis diagnosis was five days.

Some studies have explored the factors associated with delays in TB treatment (Cai et al., 2015; Rattananupong et al., 2015; Williams et al., 2018). In a systematic review and meta-analysis of 45 studies, male patients and extended travel times/distances to the initial healthcare provider were associated with shorter patient and provider delays (Cai et al., 2015). In addition, unemployment, low income, haemoptysis, and positive sputum smears were associated with patient delay (Cai et al., 2015). Another study involving 133 patients reported that cough and hospital admission were associated with shorter health system delays, while age 65 or older was associated with longer delays (Williams et al., 2018).

In a retrospective study involving 239,532 patients, Wallace (2008) found that TB prevalence and trends were comparable in metropolitan and regional areas. In a similar study, including 16,784 patients, Abubakar reported that 45% of cases did not complete therapy in rural areas, compared to 26% of cases in urban areas.

TB incidence in Victoria remains low, with 436 TB cases notified in 2018, representing 6.9 cases per 100,000 population (Bright et al., 2020). In Australia, research to date has tended to focus on metropolitan areas, where case numbers typically predominate. Understanding TB treatment delays among regional patients provides important insights into VTP performance and is a critical step towards tuberculosis elimination. Globally, TB surveillance data have been recognised as an important data source for assessing the disease burden and epidemiological trends in TB (World health organisation, 2022). Evaluating treatment outcomes and delays in regional areas will inform practice and policy. We aimed to describe notified TB cases in regional Victoria from 1995 to 2019, including trends and outcomes over these 24 years.” Lines 56-75.

Your feedback 2. The authors have run two different models and identified four factors (three in the logistic model and one in the Cox model). However, they did not discuss these factors in detail (or not available) in the discussion section.

Our feedback. We have expanded on discussion regarding these points, including adding the following statement to the discussion section: “Our analysis indicates that regionality is not an independent determinant of dying of TB. This is inconsistent with a study by Mutembo et al. (29), which reported that rural locations in Zambia had a 70% higher risk of death; however these differences have occurred in the context of our higher resourced and lower incidence setting. We did identify that that older age and male sex were predictors of lower treatment completion in regional Victoria, which may allow for interventions in our context. In particular, awareness of lower rates of treatment completion in these groups can be considered in assessing the level of follow up support and medication supervision required for people with TB, and encourage targeted education and adherence support services to groups with observed lower rates of completion.” Lines 469-478.

Reviewer #4: Comments to the Author

Your feedback 1. The authors did described the research questions (trend, and difference of regionality on outcomes), and statistical analysis conducted. But I do not see how the statistical approach were used to answer each question. For example, the author provided trend chart to describe the trend but failed to describe in the methods section/statistical analysis section that he did this. I did not realize the author used a trend chart until I read the results section.

Our response. The following statement has been added to the analysis section: “We compared the number of TB cases and the TB incidence rates in regional and metropolitan areas from 1 January 1995 to 31 December 2019. Trends over this period were displayed graphically. Data were considered in 5 year increments, with the total number of notifications per 5 year category serving as the denominator. Given the recognised connection between age and risk of tuberculosis, we also stratified case notification by age for examination of overall trends.” Lines 226-231.

Your feedback 2. Secondly, the author in many places at the results section described “stable, increase, decrease” without statistical testing the data. Visually see the point estimation in a trend chart is not strong enough to make such conclusion. The author should at least add error bar in the fig and/or describe statistical tests results in the text.

Our response. Figure 2 reports the number of reported cases, there is no inferring. We are not intending to show significance, just commenting on the general trends observed in the graph. We have changed the statement and, it now reads “In the overseas-born cohort, 29 people were under the age of 20, 236 were between the ages of 20 and 49, and 136 were ≥50 (Fig 2). Twenty cases were under the age of 20, 43 were aged 20–49 years, and 137 were ≥50 in the Australian-born cohort. TB cases among overseas-born people aged 20-49 years increased from 22 to 79 cases between 2000 and 2019. Conversely, Australian-born cases in this age bracket remained effectively unchanged over the same period (11 cases vs 10 cases).” Lines 321-326.

Your feedback 3. The authors described chi-square used but I did not see any chi-square results.

Our feedback. The statement “Pearson’s x^2^ test was used to test the association between categorical variables” was deleted. Line 234.

Your feedback 4. It seems to me the author collapsed the age groups in to <65 and >65 in alter analysis, but this is not described anywhere. In fact, in table 1, I see a more granulated age category. This is not consistency throughout.

Our response. We added the following statement to the analysis section: “We chose a more granular age category in Table 1 to describe the patients to determine which age group is impacted more by tuberculosis in order to plan for the targeted TB management program. The age groups < 65 years and ≥ 65 years were used in the univariate and multivariate analysis because, in some studies, the age groups ≥ 65 years have been found to be associated with poor treatment outcomes (Vasankari et al., 2007; Kang et al., 2021).” Lines 229-233.

Your feedback 5. Similar to the issued to the last bullet, the author collapsed multiple years of data when presenting the trend chart, it is fine to do so but the author did not describe this in any section.

Our feedback. We have addressed this point in response to feedback 1. Lines 224-228.

Your feedback 6. The flow chart should be included in the methods/study sample section rather than the results section

Our feedback. We followed the Strengthening The Reporting of Observational Studies in Epidemiology (STROBE) guidelines. According to the guidelines the flow chart is in results section (Von Elm, et al., 2007). Line 275.

Your feedback 7. The study did not clearly define what are the variables included in the study and why they are included. For example, died of TB clearly is not an independent variable, even if it is not the primary interested outcome (although I think they are important outcome variables). They should not be described as independent variables (line 164-166).

Our response. We amended the variable section, and the new section reads:

*“Variables*

The variables in this study included patient outcomes and predictors of the outcomes. The study outcomes were dependable variables: treatment outcomes and treatment delays. Treatment outcomes included completed treatment, lost to follow-up, died of TB, died of other causes during treatment for TB, and being transferred interstate or overseas.

Patients were defined as having died of TB when the clinical mode of death, the severity of TB disease (based on the presence of systemic symptoms, the extent of involvement of affected organs and central nervous system involvement), the presence of massive haemoptysis or respiratory, multisystem, or specific vital organ failure could be linked to tuberculosis and no other likely cause (Walpola, 2003). Died from other causes was defined as a death that was not attributed to tuberculosis based on pathological or autopsy examinations and death certificates indicating that the cause of death was another disease (Walpola, 2003). Patients were defined as having completed treatment if they had completed a minimum of 6 months of therapy and assessed as having completed treatment by the treating physician (Dale et al., 2016). Lost to follow-up was a treatment outcome for patients who did not complete therapy because they could not be located (Jasmer et al., 2004). Because of a change of residency, some patients were transferred interstate or overseas to continue their treatment.

For the treatment delays, we adapted the definitions outlined by Van Wyk et al. (2011), which proposed that: ‘Patient treatment delay’ is the period (in the number of days) between the onset of any self-reported TB symptoms and the first visit to a health care facility. ‘Health system delay’ is the period (in the number of days) between the first healthcare facility visit and initiation of TB treatment. ‘Diagnostic delay’ is defined as the period (in the number of days) between the onset of any self-reported TB-related symptoms and the time a chest x-ray was performed. ‘Treatment initiation delay’ was the period between a positive specimen (TB confirmed) and treatment initiation.

We extracted the following independent variables: (1) demographic data: age (age groups in years), sex (male or female), country of birth (name of the country), Aboriginal and Torres Strait Islander status (Aboriginal and/or Torres Strait Islander or not Aboriginal and/or Torres Strait Islander), local government areas (local government area), self-reported residency status (Australian-born, permanent resident, refugee/humanitarian, visitor, overseas student, other and unknown status), and for overseas-born cases, year of arrival in Australia (year). (2) Clinical characteristics: year of tuberculosis notification (year), the manifestation of tuberculosis (pulmonary, extrapulmonary or both), chest X-ray results (abnormal, cavitation or normal), laboratory results (smear, culture, or gene expert). Most of these variables have been reported as influencing patient outcomes in some studies (Dale et al., 2016; Kang et al., 2021). Lines 166-199.

Your feedback 8. The author described that they included all the covariates that are available in the multivariable models because “they could all affect the outcomes (line 198)”, which does not seem to be a sound approach, the authors should either find literature support or conducted bivariate analysis to confirm that those are covariates included

Our feedback. We added the following statement to the analysis section: Area of residence, age, sex, drug susceptibility, and country of birth were included in univariable as well as multivariable analysis because there were found to affect TB treatment outcomes in some studies (Dale et al., 2016; Kang et al., 2021; Mukherjee et al., 2012). Lines 239-241.

Your feedback 9. For survival analysis specifically, I agree with one of the reviewers who reviewed this paper before “the methods section of the abstract is quite brief missing important details that will help readers to understand the results presented from a survival analysis. Sample size, follow-up, date variables, outcomes, censoring, and statistical analysis issues are missing”. For time to event analysis, especially survival analysis, at least the reader needs to know how the authors calculate the time interval, when is the starting point of the time interval and what censoring approaches used (i.e., right censoring, left censoring). How many have the event (outcome = 1, how many lost to follow-up). The author added some language to respond to this comment but I don’t thin it fully addressed the concern.

Our response: We added the following statement to the abstract: “The time follow-up for patient delay started at symptom onset date, and the event was the presentation to the healthcare centre. For healthcare system delay, follow-up time started at the presentation to the healthcare centre, and the event was, commenced on TB treatment.” Lines 39-42.

We added the following statement to the analysis section: “We described the follow-up periods as follows: The time follow-up for patient delay started at symptom onset date, and the event was the presentation to the healthcare centre. For healthcare system delay, follow-up time started at the presentation to the healthcare centre, and the event was, commenced on TB treatment. Diagnostic delay: follow-up time began at the presentation to the healthcare centre, and the event was the first chest x-ray examination. Treatment delay: follow-up time started at first abnormal chest X-ray examination, and the event was, commenced on TB treatment. Treatment delay 2: follow-up began on the first date the patient had a positive specimen for TB, and the event, was commenced on TB treatment. Censoring occurred when patients failed to have an event because they either transferred interstate/overseas or dies or lost to follow-up (right censor).” Line 253-263.

Your feedback 10: How many have the event (outcome = 1, how many lost to follow-up). The author added some language to respond to this comment but I don’t thin it fully addressed the concern.

Our feedback. We have added the following information and a table to the results section: “

Table 5 shows patients who did not have an event in the survival analysis. Among 1,343 patients with pulmonary TB in the metropolitan area, 161 did not undergo chest x-ray examination after their first presentation to the health care centre compared to 16 out of 122 in the rural area. Nine out of 1,107 people in metropolitan with extrapulmonary TB did not commence treatment after their first presentation to the healthcare centre compared to none in rural areas. The main reasons for not having an event in the treatment delay analysis were lost to follow-up, died of another cause, or died of TB, or transferred interstate or overseas before the event. For example, in health system delay for patients with pulmonary involvement, in the rural cohort, one patient died from another cause, two died of TB and one transferred interstate or overseas after first presenting to the health care centre but before commencing TB therapy. In the metropolitan cohort, one was lost to follow-up, eight died from another cause, 12 died of TB, and 17 transferred interstate or overseas before commencing treatment.”

**Table 5. Patients who did not have an event in the survival analysis**

|  | Patients who did have an event | Patients who did not have an event | Patients who did have an event | Patients who did not have an event |
| --- | --- | --- | --- | --- |
| Patients with pulmonary involvement | Rural |  | Metropolitan |  |
| Patient delay. Event: attending health centre after experiencing TB symptoms | 113 | 0 | 1,293 | 0 |
| Health system delay. Event: starting treatment after first health care centre visit | 146 | 4 | 1,691 | 38 |
| Diagnosis delay. Event: having chest x-ray after the first presentation to the health care centre | 122 | 16 | 1,343 | 161 |
| Treatment delay: Event: starting tuberculosis treatment after undergoing first chest x-ray | 131 | 3 | 1,565 | 21 |
| Extrapulmonary patients |  |  |  |  |
| Patient delay. Event: attending health centre after experiencing TB symptoms | 62 | 0 | 907 | 0 |
| Health system delay. Event: starting treatment after first health care centre visit | 78 | 0 | 1,107 | 9 |
| Diagnosis delay. Event: having chest x-ray after the first presentation to the health care centre | 47 | 22 | 798 | 218 |
| Treatment delay 2. Event: treatment initiation after first positive TB specimen test | 151 | 7 | 1,996 | 51 |

Your feedback 11. One last concern I had is on table 1 where cells with small counts were presented. I am not know the specific rule in Australia for reporting patient data. But if you request/download any public health data from US CDC, you’ll notice that any cross-tabulated data would mask the cells with small counts. This is because by combining multiple PHIs, we can easily identify individual patients if it presents in the small enough cell. I would recommend the author either mask those cells or collapse them into less granulated categories.

Our feedback: Given that the data presented in Table 1 covers a wide geographic region and a 25 year period of time, we consider that the risk of patient identification in this data set is low. To avoid any perceived issues with confidentiality, after review of this table we have collapsed the row for contemporaneous treatment (n=1) into the ‘unknown outcome’ category.

**Reference:**

Abubakar, I., Crofts, J.  p., Gelb, D., Story, A., Andrews, N., & Watson, J.  m. (2007). Investigating urban–rural disparities in tuberculosis treatment outcome in England and Wales. Epidemiology and Infection, 136(1), 122–127. <https://doi.org/10.1017/S0950268807008333>

Bright, A., Denholm, J., Coulter, C., Waring, J., & Stapledon, R. Tuberculosis notifications in Australia, 2015-2018. Commun Dis Intell. 2020;44:1–39.

Cai, J., Wang, X., Ma, A., Wang, Q., Han, X., Li, Y. (2015). Factors associated with patient and provider delays for tuberculosis diagnosis and treatment in asia: a systematic review and meta-analysis. PLoS ONE [Electronic Resource], 10, e0120088. <https://dx.doi.org/10.1371/journal.pone.0120088>

Dale, K., Tay, E., Trauer, J. M., Trevan, P., & Denholm, J. (2017). Gender differences in tuberculosis diagnosis, treatment and outcomes in Victoria, Australia, 2002-2015. The International Journal of Tuberculosis and Lung Disease, 21(12), 1264–1271. <https://doi.org/10.5588/ijtld.17.0338>

Dale, K., Tay, E., Trevan, P., & Denholm, J. T. (2016). Mortality among tuberculosis cases in Victoria, 2002-2013: case fatality and factors associated with death. The International Journal of Tuberculosis and Lung Disease, 20(4), 515–523. <https://doi.org/10.5588/ijtld.15.0659>

Jasmer, R. M., Seaman, C. B., Gonzalez, L. C., Kawamura, L. M., Osmond, D. H., & Daley, C. L. (2004). Tuberculosis Treatment Outcomes: Directly Observed Therapy Compared with Self-Administered Therapy. American Journal of Respiratory and Critical Care Medicine, 170(5), 561–566. <https://doi.org/10.1164/rccm.200401-095OC>

Kang, Y., Jo, E. J., Eom, J. S., Kim, M. H., Lee, K., Kim, K. U., Park, H. K., Lee, M. K., & Mok, J. (2021). Treatment Outcomes of Patients with Multidrug-Resistant Tuberculosis: Comparison of Pre- and Post-Public-Private Mix Periods. *Tuberculosis and respiratory diseases*, *84*(1), 74–83. <https://doi.org/10.4046/trd.2020.0093>

Kelly, A. M., D'Agostino, J. F., Andrada, L. V., Liu, J., Larson, E. (2017). Delayed tuberculosis diagnosis and costs of contact investigations for hospital exposure: new york city, 2010-2014. American Journal of Infection Control, 45, 483-486. <https://dx.doi.org/10.1016/j.ajic.2016.12.017>

Mukherjee, A., Saha, I., Sarkar, A., & Chowdhury, R. (2012). Gender differences in notification rates, clinical forms and treatment outcome of tuberculosis patients under the RNTCP. *Lung India, 29*(2), 120-122. doi:https://doi.org/10.4103/0970-2113.95302

Mutembo, S., Mutanga, J. N., Musokotwane, K., Kanene, C., Dobbin, K., Yao, X., Li, C., Marconi, V. C., & Whalen, C. C. (2019). Urban-rural disparities in treatment outcomes among recurrent TB cases in Southern Province, Zambia. BMC Infectious Diseases, 19(1), 1087–1087. <https://doi.org/10.1186/s12879-019-4709-5>

Rattananupong, T., Hiransuthikul, N., Lohsoonthorn, V., Chuchottaworn, C. (2015). Factors associated with delay in tuberculosis treatment at 10 tertiary level care hospitals in thailand. Southeast Asian Journal of Tropical Medicine & Public Health, 46(4), 689-96. Retrieved from <http://ovidsp.ovid.com/ovidweb.cgi?T=JS&PAGE=reference&D=med12&NEWS=N&AN=26867389>.

Van Wyk SS, Enarson DA, Beyers N, Lombard C, Hesseling AC. Consulting private health care providers aggravates treatment delay in urban South African tuberculosis patients. Int J Tuberc Lung Dis. 2011;15(8):1069–76. Imperial, M. Z., Nahid, P., Phillips, P. P., Davies, G. R., Fielding, K., Hanna, D., ... & Savic, R. M. (2018). A patient-level pooled analysis of treatment-shortening regimens for drug-susceptible pulmonary tuberculosis. *Nature medicine*, *24*(11), 1708-1715.

Vasankari, T., Holmström, P., Ollgren, J., Liippo, K., Kokki, M., & Ruutu, P. (2007). Risk factors for poor tuberculosis treatment outcome in Finland: a cohort study. BMC Public Health, 7(1), 291–291. <https://doi.org/10.1186/1471-2458-7-291>

Von Elm, E., Altman, D. G., Egger, M., Pocock, S. J., Gøtzsche, P. C., & Vandenbroucke, J. P. (2007). The Strengthening the Reporting of Observational Studies in Epidemiology (STROBE) Statement: Guidelines for Reporting Observational Studies. Epidemiology (Cambridge, Mass.), 18(6), 800–804. <https://doi.org/10.1097/EDE.0b013e3181577654>

Wallace, R. M., Armstrong, L. R., Pratt, R. H., Kammerer, J. S., & Iademarco, M. F. (2008). Trends in Tuberculosis Reported From the Appalachian Region: United States, 1993-2005. The Journal of Rural Health, 24(3), 236–243. <https://doi.org/10.1111/j.1748-0361.2008.00164.x>

Walpola, H. C., Siskind, V., Patel, A. M., Konstantinos, A., & Derhy, P. (2003). Tuberculosis-related deaths in Queensland, Australia, 1989-1998: characteristics and risk factors. The International Journal of Tuberculosis and Lung Disease, 7(8), 742–750.

Williams, E., Cheng, A. C., Lane, G. P., & Guy, S. D. (2018). Delays in presentation and diagnosis of pulmonary tuberculosis: a retrospective study of a tertiary health service in Western Melbourne, 2011–2014. Internal Medicine Journal, 48(2), 184–193. <https://doi.org/10.1111/imj.13551>

World Health Organisation. Strengthening TB surveillance. [Internet]. 2022 [cited 2022 Jun 26]. Available from: <https://www.who.int/westernpacific/activities/strengthening-tb-surveillance>

Yimer, S. A., Bjune, G. A., & Holm-Hansen, C. (2014). Time to first consultation, diagnosis and treatment of TB among patients attending a referral hospital in Northwest, Ethiopia. *BMC infectious diseases*, *14*, 19. <https://doi.org/10.1186/1471-2334-14-19>
